# Molecular phenotype of zebrafish ovarian follicle by serial analysis of gene expression and proteomic profiling, and comparison with the transcriptomes of other animals

**DOI:** 10.1186/1471-2164-7-46

**Published:** 2006-03-09

**Authors:** Anja Knoll-Gellida, Michèle André, Tamar Gattegno, Jean Forgue, Arie Admon, Patrick J Babin

**Affiliations:** 1 Génomique et Physiologie des Poissons, UMR NUAGE, Université Bordeaux 1, 33405 Talence cedex, France; 2 Department of Biology, Technion-Israel Institute of Technology, Haifa 32000, Israel

## Abstract

**Background:**

The ability of an oocyte to develop into a viable embryo depends on the accumulation of specific maternal information and molecules, such as RNAs and proteins. A serial analysis of gene expression (SAGE) was carried out in parallel with proteomic analysis on fully-grown ovarian follicles from zebrafish (*Danio rerio*). The data obtained were compared with ovary/follicle/egg molecular phenotypes of other animals, published or available in public sequence databases.

**Results:**

Sequencing of 27,486 SAGE tags identified 11,399 different ones, including 3,329 tags with an occurrence superior to one. Fifty-eight genes were expressed at over 0.15% of the total population and represented 17.34% of the mRNA population identified. The three most expressed transcripts were a rhamnose-binding lectin, beta-actin 2, and a transcribed locus similar to the H2B histone family. Comparison with the large-scale expressed sequence tags sequencing approach revealed highly expressed transcripts that were not previously known to be expressed at high levels in fish ovaries, like the short-sized polarized metallothionein 2 transcript. A higher sensitivity for the detection of transcripts with a characterized maternal genetic contribution was also demonstrated compared to large-scale sequencing of cDNA libraries. Ferritin heavy polypeptide 1, heat shock protein 90-beta, lactate dehydrogenase B4, beta-actin isoforms, tubulin beta 2, ATP synthase subunit 9, together with 40 S ribosomal protein S27a, were common highly-expressed transcripts of vertebrate ovary/unfertilized egg. Comparison of transcriptome and proteome data revealed that transcript levels provide little predictive value with respect to the extent of protein abundance. All the proteins identified by proteomic analysis of fully-grown zebrafish follicles had at least one transcript counterpart, with two exceptions: eosinophil chemotactic cytokine and nothepsin.

**Conclusion:**

This study provides a complete sequence data set of maternal mRNA stored in zebrafish germ cells at the end of oogenesis. This catalogue contains highly-expressed transcripts that are part of a vertebrate ovarian expressed gene signature. Comparison of transcriptome and proteome data identified downregulated transcripts or proteins potentially incorporated in the oocyte by endocytosis. The molecular phenotype described provides groundwork for future experimental approaches aimed at identifying functionally important stored maternal transcripts and proteins involved in oogenesis and early stages of embryo development.

## Background

Folliculogenesis and oogenesis include the formation of ovarian follicles, the initiation and completion of meiosis, and the accumulation of specific information and molecules such as RNAs, proteins, or imprinted genes in the female germ cells to sustain embryo development to the stage where zygotic gene activation takes over [1-3].

The zebrafish, *Danio rerio*, is currently the most popular fish model in developmental and genomic analyses and a genome sequencing project is currently underway [4]. A large number of expressed sequence tags (ESTs) is already available [5]. A number of methods are currently used for gene expression profiling. They differ in scale, economy, and sensitivity. Delineation of the transcriptome of teleost fish ovaries has been evaluated using large-scale EST sequencing [6] of cDNA libraries of zebrafish [7] and Atlantic salmon [8,9] or subtractive hybridisation of cDNA libraries [10] of medaka [11] or rainbow trout [12] gonads. While these methods give an idea of transcript abundance or enrichment in a specific tissue, a few genes expressed at high levels usually represent a large proportion of the total transcripts and are thus more frequently represented in the EST database [13].

Serial analysis of gene expression (SAGE) based on the enumeration of directionally reliable short cDNA sequences (tags), provides qualitative as well as quantitative analysis of a large number of genes in a defined tissue [14,15]. This is a method of choice for discovering novel genes and spliced variants. This technique has been widely applied in human studies and various SAGE tags/SAGE libraries have been generated from different cells/tissues, including human oocytes [16,17], thus enabling the successful identification of differentially expressed genes in normal physiological processes and pathological conditions [18].

The aim of this work was to profile the transcriptome of fully-grown zebrafish follicles using the SAGE method and compare it with the protein repertoire determined at the same stage of oogenesis after one- (1D) and two-dimensional (2D)-polyacrylamide-gel electrophoresis (PAGE) protein fractionation and in-gel proteolysis, followed by tandem mass spectrometry (MS/MS) identification of the resulting peptides [19]. The database generated was compared with several vertebrate ovary/unfertilized egg transcriptomes generated with the large-scale EST sequencing approach in order to identify functionally important maternal transcripts and proteins stored in germ cells.

## Results

### Zebrafish fully-grown follicle transcriptome

**Figure 1:**
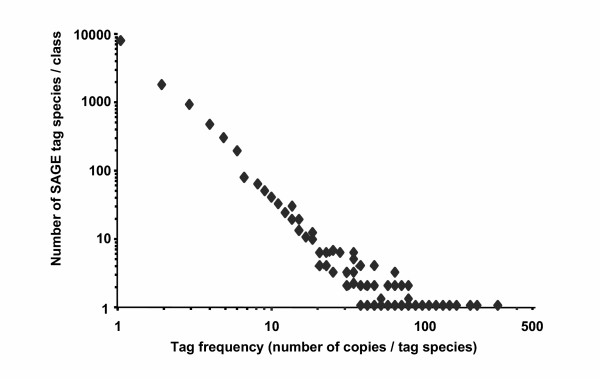
**Distribution of ZEBRAOV library SAGE tags by abundance classes**. The library was subdivided into 83 abundance classes, from 1 to 284 tag copies per tag species, according to the occurrence frequency of each tag species. Tag frequencies were plotted on a logarithmic scale on the x-axis against the number of SAGE tag species in each class on the y-axis.

An essential step in SAGE library analysis is the unambiguous assignment of each SAGE tag to the corresponding mRNA or EST sequence. This tag-to-gene mapping requires an initial *in silico *SAGE tag extraction of virtual tags found in public EST/cDNA sequence databases. Existing web sites [20-23] provide the correspondence between SAGE tags and transcripts. A limited number of species have been subjected to SAGE analysis, as seen on the SAGEmap web site [20,24], which presents SAGE tag to UniGene mapping for eighteen species. As no previous study had used the SAGE strategy in any fish species, there was no *in silico *SAGE tag database available for zebrafish. A generic computer package named FISHTAG was designed to extract virtual SAGE tags from UniGene and TIGR EST databases and generate a zebrafish *in silico *SAGE tag database, ZEBRATAG. Applying SAGE to the zebrafish fully-grown ovarian follicle generated a catalogue of 27,486 sequenced tags, ZEBRAOV. The list of these experimental SAGE tags and their relative frequencies were deposited in the NCBI gene expression and hybridization array data repository (GEO) [25] under accession number GSE3679 (Additional file 1: Table 1). Analysing these tags led to the identification of 11,399 unique transcripts, including 3,329 tags with an occurrence superior to one. ZEBRAOV was subdivided into eighty-three abundance classes, from 1 to 284 tag copies per tag species (Figure 1), according to frequency of occurrence.

Around 87% (2,898) and 67% (2,223) of ZEBRAOV non-singleton tags were assigned using FISHTAG to at least one UniGene (Build#87) or TIGR (release 16.0) cluster, respectively. Fifty-eight transcripts, identified by their SAGE tags, were expressed at over 0.15%, i.e. the number of times a tag was counted in ZEBRAOV was at least 42, (Table 1), accounted for 17.34% of the mRNA population identified and represented forty-six of the most abundant classes (Figure 1). Among these SAGE tags, thirty-seven were assigned, using the FISHTAG software package with UniGene database Build#87 as a reference, to at least one UniGene cluster in a correct position of sense (R1) or antisense (R1cr) transcript sequences, which were annotated or not. Nineteen tags were multiple-matched, i.e. matching over one UniGene cluster. Two tags did not match any reference cDNA sequences in GenBank™/EBI Data Bank after extraction of their SAGE tags at the first three positions and could not, thus, be classified. Unmatched SAGE tags in the SAGE library could be due to the presence of genes in the zebrafish genome or spliced variant transcripts that had not previously been identified by EST data. Some of these unidentified transcripts were widely expressed in the zebrafish fully-grown follicle transcriptome (see Table 1).

The most abundant tag in ZEBRAOV was recovered 284 times, thus representing 1.033% of the mRNA population evaluated by the SAGE method (Table 1). This SAGE tag was identified four times on chromosome 22 genomic contig (GenBank (gb) accession number gb|NW_634208|) and in two deduced transcript variants, gb|XM_702755| and gb|XM_704598|, the latter being nearly identical to the consensus sequence of full-length ovary cDNA entries gb|BM141044| and gb|CO351300|. These transcripts are part of a large conserved protein family with at least thirteen transcript variants centred on locus LOC561392 of chromosome 22. Deduced protein sequence gb|BM141044| was 41.6% identical in 125 amino acid overlap with the N-terminal part of human latrophilin-2 precursor protein (UniProt (up) accession number up|O95490|). It contains the galactose/rhamnose-binding lectin domain found in numerous proteins with sugar binding properties (Pfam accession number PF02140) [26], including the two domains found in rhamnose-binding lectins in catfish (up|Q9PVW8|) and rainbow trout (up|Q9IB51|, up|Q9IB52|, up|Q9IB53|) eggs. It should be noted that UniGene (ug) cluster number ug|Dr.12439| enclosing SAGE tag N°1 was in fact a chimera cluster also containing EST sequences from heterogeneous nuclear ribonucleoprotein K (*hnrpk*) transcript gb|NM_212994| and, therefore, could not be used as a valid reference UniGene cluster. *hnrpk *is in fact located on chromosome 8 in the zebrafish genome (Ensembl Gene ID ENSDARG00000018914).

Components of the cytoskeleton, β-actin 2 (*bactin2*) (SAGE tag N°2), β-actin 1 (*bactin1*) (SAGE tag N°12), tubulin β-2 (*tubb2*) (SAGE tag N°43), and a transcript similar to thymosin beta 10 (SAGE tag N°14), as well as claudin d (*cldnd*) (SAGE tag N°7), a constituent of the tight junction complex, were among the most highly-expressed transcripts. A simultaneous high expression of different isoforms of several zona pellucida (ZP) glycoprotein transcripts was also observed with common (SAGE tag N°15, *zp2.3 *or *zp2.4 *variant proteins) or distinct (SAGE tag N°19, *zp3b*, and SAGE tag N°20, *zp3*) tags.

A transcribed locus (gb|XM_701504|) similar to the H2B (*h2b*) histone family and derived from an annotated genome sequence (gb|NW_649547|) contained the third most abundant annotated SAGE tag in the correct sense position. The corresponding UniGene cluster ug|Dr.46793| was a chimera cluster, while the correct EST cluster of this transcribed portion of the zebrafish genome was located in TIGR (TC291160). We also observed that a set of eight transcripts for genes associated with S-, L-, and P0-type ribosomal proteins, individually expressed at over 0.15%, represented a total of 1.63% of the total follicle transcripts evaluated by the SAGE method (Table 1).

SAGE tag N°5, with 142 tags sequenced, representing 0.517% of the mRNA population, was found in the correct sense position of the short (561 bp) transcript (gb|BC049475|) of metallothionein (*mt2*) on chromosome 18 (Ensembl Gene ID ENSDARG00000041553). A longer (1,739 bp) *mt2 *transcript (gb|NM_194273|) was recovered from the same UniGene cluster, ug|Dr.47289|. However, the corresponding SAGE tag (CATGAGTGTGAGAT) from this long transcript was different from that of the short (CATGTCCTTTGTCT) transcript and was not detected in ZEBRAOV. In addition to the *mt2 *short transcript, another abundant transcript related to metal binding, ferritin heavy polypeptide 1 (*fth1*) (SAGE tag N°46, gb|BC045278|), was also detected with a SAGE tag occurrence of 51. Analysis of ZEBRAOV revealed the presence of an additional highly-expressed tag with an occurrence of 23, linked to a transcript similar to the ferritin heavy subunit found in zebrafish embryos (gb|CB360372|, gb|AI883498|) and testes (gb|BI672352|).

SAGE tag N°8 was part of an abundant transcript (gb|NM_199781|) encoding LOC327299, a hypothetical protein on zebrafish chromosome 3 (Dr.47296, zgc:66160) with similarities to another zebrafish transcript (gb|XP_691610|), itself similar to the *Xenopu*s protein MGC68553 (gb|AAH60006|). The deduced proteins have high sequence similarities with a mammalian deduced protein of unknown function (e.g. human hypothetical protein LOC29035, gb|NP_054836|, and bovine CG4768-PA isoforms, gb|XP_611066|). Another abundant tag (SAGE tag N°24) was assigned to a transcribed locus, while no significant sequence similarity was detected in any annotated sequence in GenBank™/EBI Data Bank. However, significant similarity was detected with other expressed transcripts from teleost fish species *Pimephales promelas *(gb|DT169547|), *Oncorhynchus mykiss *(gb|CX039674|), and *Salmo salar *(gb|CA037218|).

SAGE tag N°9 was included in cDNA EST clones (e.g. gb|BM861564|), which contains repetitive element MSR1 in addition to a transcript similar to ZP1-related protein (gb|XM_686880|). This tag was recovered at least three times in a genome sequence of linkage group 11 (gb|BX548064|).

The heat shock protein 90-beta (*hsp90b*) (SAGE tag N°10) transcript was also remarkably expressed, together with a locus similar to stress-associated endoplasmic reticulum protein 1 (SAGE tag N°16), a cold inducible RNA binding protein (*cirbp*) (SAGE tag N°52), and 40 S ribosomal protein S27a, similar to ubiquitin (SAGE tag N°40).

The gene products for the following enzymes: lactate dehydrogenase B4 (*ldhb*) (SAGE tag N°11), subunit 9 of ATP synthase of mitochondrial F0 complex (*atp5g*) (SAGE tag N°13), retinol dehydrogenase 10 (*rdh10*) (SAGE tag N°28), a protein similar to cytochrome oxidase III (SAGE tag N°32), glutathione S-transferase pi (*gstp1*) (SAGE tag N°35), and a proteolytic enzyme similar to cathepsin S (SAGE tag N°41) were also recovered.

The UniGene clusters identified in ZEBRAOV using tags at R1 or R1cr positions and expressed in over 0.05% of the total transcript population of fully-grown follicles were classified according to the Gene Ontology (GO) [27] system, with help of updated information from the zebrafish information network (ZFIN) database [28]. The distribution by molecular function GO terms was: 32.6% binding properties, including 22.2% nucleic acid binding; 11.1% structural molecule activity; 10.4% catalytic activity; 3.5% transporter activity; 2.1% signal transducer activity; 1.4% translation regulator activity; 0.7% translation initiation factor activity; and 0.7% enzyme inhibitor activity, while the remaining 37.5% have an unknown postulated molecular functions. In addition to molecular function categories, ZEBRAOV was used to identify transcript members of specific biological processes or metabolic pathways. For example, the following transcripts were identified in ZEBRAOV by their tags in R1 or R1cr position and related to the cell cycle: cyclin A1 (*ccna1*) (SAGE tag N°38, Table 1), a transcribed locus moderately similar to zygote arrest 1 (SAGE tag N°21, Table 1), cyclin B1 (*ccnb1*, tag occurrence 25, gb|NM_131513|), cyclin B2 (*ccnb2*, tag occurrence 13 for a long transcript, gb|BC045937| and tag occurrence 4 for a short transcript, gb|AF365872|), cyclin G1 (*ccng1*, tag occurrence 3, gb|BC052125|), cyclin-dependent kinase 9 (*cdk9*, occurrence 3, gb|NM_212591|), and cyclin L1 (*ccnl1*, tag occurrence 2, gb|NM_199740|). As a second example, some of the transcripts encoded proteins related to the lipoprotein or fatty acid metabolisms, such as low density lipoprotein receptor-related protein associated protein 1 (*lrpap1*, tag occurrence 4, gb|BC049517|), high density lipoprotein-binding protein (vigilin) (*hdlbp*, tag occurrence 4, gb|AI545520|), fatty acid binding protein 3 (*fabp3*, tag occurrence 4, gb|NM_152961|), and fatty acid binding protein 7b (*fabp7b*, tag occurrence 2, gb|AY380814|).

### Comparison of ZEBRAOV with zebrafish ovary cDNA libraries

**Figure 2:**
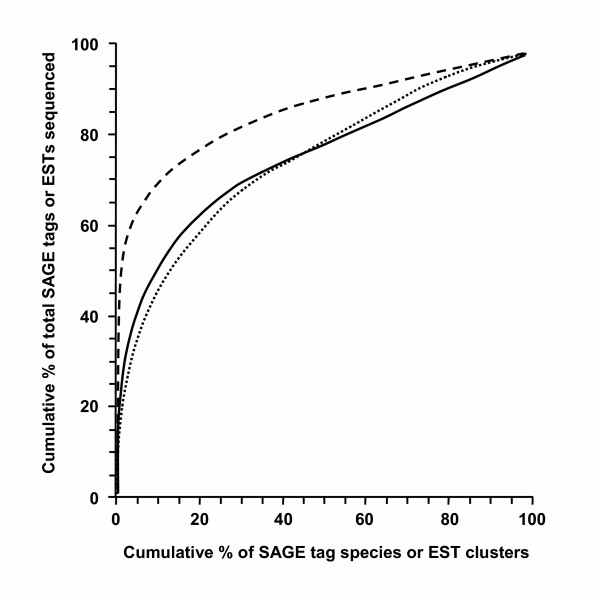
**Transcriptional activity of zebrafish fully-grown follicles and ovaries evaluated by the composition of the ZEBRAOV SAGE tag database and EST clusters in two selected cDNA libraries**. The cumulative percentage of tag species in the SAGE tag library (solid line) or number of EST clusters in cDNA libraries ID.9767 (dotted line) and ID.15519 (dashed line) (representing the number of transcripts identified), were plotted in order of abundance on the x-axis against the cumulative percentage of the corresponding total tag or EST counts (representing the total amount of sampled transcriptome in SAGE or dbEST libraries) on the y-axis.

The cumulative percentages of SAGE tag species or EST clusters were plotted in order of abundance, according to the cumulative percentages of tag or EST counts, providing a comparative view of transcriptional activity (Figure 2). A similar proportion of transcripts was observed between ZEBRAOV and cDNA library ID.9767, while library ID.15519 contained a few clusters with an over-representation of their EST numbers. At 50% of the cumulative percentage of tags or ESTs, there were 9% ZEBRAOV SAGE tags, numbered from 1 to 1026, and 12.5% ID.9767 EST clusters, numbered from 1 to 348. In contrast, in library ID.15519, the majority of the ESTs sequenced originated from a small number of unique transcripts, i.e. 1% of the total clusters, numbered from 1 to 26, and represented 50% of the ESTs in the library. The differences observed between the proportions of transcripts in the two cDNA libraries were also illustrated by the difference in the number of ESTs per transcript abundance class. Transcript numbers in libraries ID.15519 and ID.9767 ranged from one copy/unique cluster in the least abundant class to 1,714 or 287 copies/unique cluster, in the most abundant class.

The occurrence of the most abundant annotated SAGE tag was compared with their corresponding UniGene cluster representation, evaluated by the number of ESTs, in the two selected zebrafish ovary cDNA libraries (Table 2). Eight clusters, including *bactin2*, *bactin1*, *zp2.3*, *zp2.4*, *zp3b*, *zp3*, and *tubb2 *were expressed over 0.15% in the three transcriptome profiles (Table 2A). UniGene cluster accession numbers Dr.30322 of *zp3*, Dr.5628, Dr.19916 of *zp2.3*, and Dr.23439 of *zp2.4*. were overrepresented in library ID.15519, accounting for over 11% of the transcripts identified in this cDNA library. Seven clusters higher than 0.15% were in both ZEBRAOV and ID.9767, e.g.: *cldnd*, transcripts similar to rhamnose-binding lectins, H2B histone family, and cathepsin S (Table 2B). ZEBRAOV and ID.15519 had three clusters in common, i.e. ribosomal protein L3 (*rpl3*), *rdh10*, and *ccna1 *(Table 2C). Twenty transcripts expressed at over 0.15% of the total mRNA population in ZEBRAOV were recovered below this limit in the ovary cDNA libraries, while some of them were not expressed at all in library ID.15519 (Table 2D). The largest discrepancies, evaluated by the difference between the mean number of EST values obtained with the cDNA libraries and the expression level indicated by SAGE tag frequency, were observed with *mt2*, *hsp90b*, and *ldhb*. The absence of expression in library ID.15519 of some UniGene clusters found in both library ID.9767 and ZEBRAOV was illustrated with SAGE tag N°21 that was part of a transcript (gb|BM533765/XM_682436/XM_703698|) moderately similar to zygote arrest 1, which contains sequences similar to the atypical PHD motif found in the zygote arrest 1 gene from vertebrate species including zebrafish *zar1 *(gb|BM533765|). The attached UniGene cluster (ug|Dr.13590|) was highly expressed in ZEBRAOV, only moderately in library ID.9767, and not in library ID.15519. It should be noted that the zebrafish *zar1 *transcript attached to UniGene cluster number Dr.12340 was found at high levels (0.370%) in library ID.9767 but not in ZEBRAOV or ID.15519.

EST data from the two selected zebrafish ovary cDNA libraries were also compared with ZEBRAOV to determine the sensitivity for detecting transcripts with a characterized maternal genetic contribution [29] (Table 3). Using the same maternal transcripts as the target sequences, the ZEBRAOV database was around 1.8 times more sensitive, for the detection of these transcripts, than the EST method previously used to describe the zebrafish ovary transcriptome. The number of selected transcripts with a maternal factor role detected by the SAGE method was 12 out of a total of 24, compared with 7 for libraries ID.9767 and ID.15519. Only two maternal transcripts, *ccnb1 *and POU domain class 5 trf 1 (*pou5f1*), were detected in ZEBRAOV and both libraries. Two transcripts, activin receptor IIb (*acvr2b*), and pre-B-cell leukemia trf 4 (*pbx4*) were found only in ZEBRAOV, while another two, cth1 (*cth1*) and activin receptor-like kinase 8 (*alk8*), were only retained in the cDNA libraries. It should be pointed out that the higher sensitivity observed with the SAGE method was obtained with around 21 times fewer sequenced cDNA clones, i.e. 576 to generate ZEBRAOV instead of 11,344 for library ID.9767 and 13,029 for library ID.15519. However, the low-abundant maternal-effect vasa homolog (*vasa*) transcript that was not detected in ZEBRAOV or in the ID.9767 and ID.15519 zebrafish dbEST libraries was identified at low levels in library ID.9874 (ug|Dr.559|, 0.056%), even if the 3,544 EST sequences in this library have only been classified in 1,475 UniGene entries.

### Polarised distribution of metallothionein 2 transcripts and those similar to rhamnose-binding lectins in zebrafish oocyte

**Figure 3:**
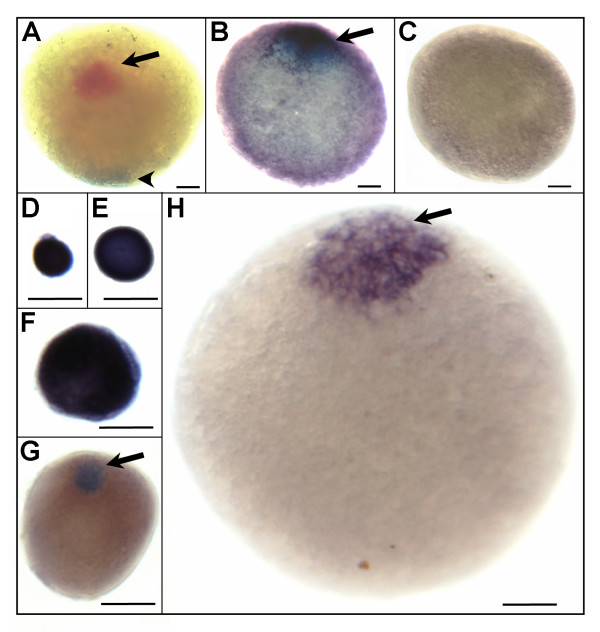
**Localisation of selected maternal transcripts in zebrafish fully-grown follicles as revealed by whole-mount *in situ *hybridization**. *A*, Polarization of the oocyte along the animal-vegetal axis was visualized using two colour whole-mount *in situ *hybridization. Cyclin B1 (*ccnb1*) mRNAs were identified at animal pole (arrow) after probe labelling with fluorescein-labelled antisense riboprobes and Deleted AZoospermia-Like (*dazl*) transcripts located at vegetal pole (arrowhead) after probe labelling with digoxigenin-labelled antisense riboprobes. The hybridization signal is coloured red with fluorescein and dark brown to blue with digoxigenin. *B*, The animal pole localisation of transcripts similar to rhamnose-binding lectin at the fully-grown follicle stage (stage IV) was detected with digoxigenin-labelled antisense riboprobes. *C*, No staining signal was observed using sense riboprobes of transcripts similar to rhamnose-binding lectin. *D-H*, Stage-specific polarised distribution of metallothionein 2 (*mt2*) short transcript isoform in stage IB (*D*, *E*), II (*F*), early stage III (*G*), and stage IV (*H*) follicles. The *met2 *hybridization signal detected at early stages was widely distributed in the ooplasma, whereas it concentrated at the animal pole from early stage III to the end of vitellogenesis. For stages III and IV, the animal pole, oriented toward top of page, is indicated by an arrow. Scale bar = 100 μm.

The polarization of oocytes along the animal-vegetative axis was visualized with *ccnb1 *mRNAs, identified at the animal pole, and Deleted AZoospermia-Like (*dazl*) located at the vegetative pole (Figure 3A). The *ccnb1 *transcript was SAGE tag N°106, with a tag occurrence of 25, detected in both ovary cDNA libraries (Table 3). The *dazl *transcript was SAGE tag N°3122, with a tag occurrence of 2, found only in library ID.15519 with two EST sequence entries. Whole-mount *in situ *hybridization using an RNA labelled probe that was potentially capable of hybridizing with all rhamnose-binding lectin transcripts variants (SAGE tag N°1, tag occurrence 284), due to their very high sequence conservation, revealed preferential polarization at the animal pole (Figure 3B). Whole-mount *in situ *hybridization using a specific *mt2 *(SAGE tag N°5, tag occurrence 142) riboprobe confirmed the presence of this mRNA in zebrafish ovarian follicles, although only four and six EST sequence entries were found in libraries ID.15519 and ID.9767, respectively. As with rhamnose-binding lectins, colocalization of the *mt2 *transcript with *ccnb1 *mRNA by two-colour whole-mount *in situ *hybridization demonstrated a signal restricted to the animal pole of the oocyte (data not shown). The distribution of this transcript was stage-dependent (Figure 3D–H). The hybridization signal was homogeneously distributed in stages I and II of oogenesis and restricted from early stage III to the animal pole of the oocyte.

### Comparison of ZEBRAOV with ovary/egg functional genomic data from other vertebrate and non-vertebrate species

The annotated transcripts expressed at over 0.15% in zebrafish fully-grown ovarian follicles (Table 1) that had corresponding UniGene clusters were used to search for homologous Unigene clusters in the ovary/unfertilized egg cDNA libraries currently available for seven vertebrate species (Table 4). The homologous vertebrate UniGene clusters were identified using UniGene homologous tool or found after BLAST [30] searches. Numerous genes expressed in zebrafish had homologous counterparts in the expression profile of human and *Xenopus *cDNA libraries and, to a lesser extent, in profiles available for other vertebrate species. Out of a total of thirty-five zebrafish genes, twenty-six homologous UniGene clusters were identified in humans, including seven at over 0.15% of the mRNA population. Twenty four homologous UniGene clusters were identified in *Xenopus*, including two at over 0.15% of the mRNA population. Due to the duplicated nature of the zebrafish genome, *bactin2 *and *bactin1 *were duplicated forms of the mammal *bactin *gene. These housekeeping genes, together with *tubb2*, were a common characteristic of ovarian vertebrate abundant cytoskeletal protein encoding clusters. Homologous *flh1 *transcript was remarkably expressed in all available vertebrate ovary/egg transcriptomes, with high levels in human, swine, and salmon cDNA libraries. This transcript was also recovered at high levels in the ID.16098 dog (*Canis familiaris*) ovary dbEST library (ug|Cfa.1238|, 0.264%). *hsp90b*, *ldhb*, *atp5g*, and 40S ribosomal protein S27a, were also some of the most expressed genes in the vertebrate ovaries. The other abundant ZEBRAOV transcripts recovered in at least one vertebrate species at a homologous cluster frequency >0.15% were *zp3 *or related gene *zp3b*, *cldnd*, *rpl3*, and *ccna1*. The *cirbp *transcript was also widely distributed, but in a lower relative proportion of mRNA.

The annotated transcripts of ZEBRAOV were also compared with published data obtained from human germinal vesicle (GV)-stage oocytes by PCR-SAGE [16]. This human SAGE tag library has not been deposited at the GEO database. The published short-list of human tags was checked against UniGene Build#187 using SAGEmap tools. Out of a total of 175 SAGE tags analysed, 81 were identified in R1 sense position. This updated list was compared with the homologous UniGene clusters expressed at over 0.15% in ZEBRAOV. Three homologous clusters were identified in the human oocyte catalogue, i.e. actin gamma 1 (*ACTG1*)/actin beta (*ACTB*) (ug|Hs.514581| and ug|Hs.520640|), ZP glycoprotein 3 (*ZP3*) (ug|Hs.488877|), and heat shock 90 kDa protein 1, beta (*HSPCB*) (ug|Hs.509736|). These clusters were also recovered at high levels in SAGE tag libraries deposited on SAGEmap for human ovarian cancer cell lines (e.g. GEO accession number GSM726). Comparison between the updated annotated list of human GV oocyte and ZEBRAOV clusters revealed that, in addition to *ACTG1*/*ACTB*, *ZP3*, and *HSPCB*, other homologous expressed clusters, identified by their SAGE tag at R1 sense position and expressed above 0.01% of the total expressed transcripts, had also been detected in ZEBRAOV: tubulin alpha 6 (*TUBA6*) and beta 4 (*TUBB4*), programmed cell death 5 (*PDCD5*), proliferating cell nuclear antigen (*PCNA*), barrier to autointegration factor 1 (*BANF1*), guanine nucleotide binding protein, beta polypeptide 2-like 1 (*GNB2L1*), CD9 antigen (*CD9*), and glyceraldehyde-3-phosphate dehydrogenase (GAPD) were found.

The most expressed transcripts in ZEBRAOV (Table 1), including the conserved expressed transcripts of vertebrate ovaries/unfertilized eggs (Table 4), were used to screen ovary/egg dbEST or SAGE tag libraries from non-vertebrate species. As expected, some homologous housekeeping genes, which encode ribosomal proteins or proteins responsible for cell structure, were among the most highly-expressed genes in these libraries. For example, homologous transcript of 40 S ribosomal protein S27a was retrieved from the silkworm (*Bombyx mori*) egg SAGE tag library at 0.366% of the total mRNA population, together with transcripts of 60 S ribosomal protein L3 (0.4%), and 60 S ribosomal large P0 (0.254%). In addition, transcripts for beta-tubulin were retrieved from silkworm (0.266%), nematode (*Caenorhabditis elegans*) (ug|Cel.10737|, 0.116%) and amphioxus (*Branchiostoma floridae*) (ug|Bfl.1179|, 0.051%) egg cDNA libraries. Other conserved homologous transcripts identified were related to the cell cycle, with cyclin A type in sea urchin (*Strongylocentrotus purpuratus*) (ug|Spu.227|, 0,499%), and amphioxus (ug|Bfl.5026|, 0,511%) eggs, or metal binding, with ferritin protein genes in silkworm (0.319%), and amphioxus (ug|Bfl.851|, 0.106%) eggs, or metallothionein in sea urchin (ug|Spu.96|, 0.066%) eggs. Catalytic activity transcripts for cytochrome c oxidase III in silkworm (0.228%), and lactate dehydrogenase in nematode (ug|Cel.22829|, 0.022%) eggs, homologous with transcripts expressed at over 0.15% in ZEBRAOV, were expressed in ovaries/eggs of non-vertebrate species.

### Comparison of zebrafish follicle protein repertoire deduced from SAGE with the protein repertoire isolated after proteomic analysis

The proteins extracted from fully-grown follicles were resolved by 1D-SDS-PAGE or 2D-PAGE, subjected to in-gel tryptic digestion, and analysed by MS/MS. The protein repertoire determined was then compared with the repertoire deduced from ZEBRAOV (Table 5). Forty-three out of a total of sixty proteins identified by proteomic analysis were initially retrieved using 1D-SDS-PAGE fractionation, forty-one by 2D-PAGE fractionation, and twenty-four were common to both. Potential molecular functions of the proteins identified using proteomic analysis according to GO terms were: 26% structural molecule activity, 25% binding properties, including 3% nucleic acid binding, 22% catalytic activity, 3% each for transporter, translation-regulator, and enzyme-regulator activity, 2% each for signal transducer, antioxidant, and electron transporter activity, while the remaining 12% had no postulated molecular function. The three most abundant categories were: (i) structural molecules, represented by beta-actin, tubulin, and ZP variant isoforms, as well as ribosomal proteins; (ii) binding proteins, mostly chaperonins and heat shock proteins; and (iii) proteins with a catalytic activity, mostly oxidoreductases, like acyl-Coenzyme A dehydrogenase or enolases, and transferases, like creatine kinase and pyruvate kinase. Comparison of transcriptome and proteome data indicated that forty-three proteins were recovered with a corresponding transcript identified by an experimental SAGE tag at a correct, R1 or R1cr, position. Seven proteins were also found with a corresponding tag in R2/R3 or Rn1/Rn2 position, and eight with corresponding multiple-matched R1 or R1cr tags. Comparing transcriptome and proteome also revealed a weak predictive value between mRNA and protein abundance. The MS-based protein identification approach recovered around 23% of the proteins, including *bactins*, *zp2.3*, *zp2.4*, *zp3*, *zp3b*, *tubb2*, *ldhb*, and ribosomal protein large, P0 (*rplp0*), deduced from transcripts expressed at over 0.15% and identified by an experimental SAGE tag in a correct, R1 or R1cr, position (Table 1). All the proteins identified by proteomic analysis had at least one transcript counterpart in ZEBRAOV, with two exceptions, eosinophil chemotactic cytokine (*chia*) and nothepsin (*nots*). UniGene cluster ug|Dr.831| of the *chia *transcript was detected three and five times in ovary cDNA banks ID.15519 and ID.9767, respectively. UniGene cluster ug|Dr.10788| of *nots *transcript was not detected either zebrafish cDNA library used. Zebrafish *chia *encoded a protein highly similar to zebrafish protein isoforms similar to chitinase (gb|XP_708403.1|, gb|XP_686386|) or proteins encoding by multiple chitinase genes in rainbow trout (gb|CAD59687|) and Japanese flounder (gb|BAD15061|) and, to a lesser extent, acidic mammalian chitinase precursor, e.g. up|Q9BZP6| in humans. Zebrafish *nots *encoded a protein similar to vertebrate aspartic-type endopeptidases, such as zebrafish cathepsin D (up|Q8JH28|) and human cathepsin E (up|P14091|).

Proteins synthesized by *bactin1 *and *bactin2 *were not resolved by the proteomic analysis, due to the amino acid sequence identity of these isoforms. However, corresponding differentially expressed transcripts were discriminated by a specific SAGE tag, due to divergent 3'-untranslated part sequences. On the contrary, a well conserved divergent 3'-untranslated part sequence led to a common SAGE tag identified in *zp2.3 *and *zp2.4 *transcripts, while a distinct SAGE tag was recovered with *zp2.2 *(Tables 1 and 5). The high sequence similarities of these three protein isoforms led to an unsolved protein identification on the gel map produced by proteomic analysis. Identical peptide sequences were also identified with tubulin alpha isoforms and recovered in different areas after 2D-PAGE fractionation. Sequence differences or additional identified peptides made it possible to discriminate between tubulin alpha 1, alpha 8 like 4, alpha 2, and alpha 3. In all cases, specific SAGE tags were identified for each tubulin transcript isoform, even if isoform alpha 8 like 4 was identified using a multiple-matched tag.

Furthermore, comparison of transcriptome and proteome data also revealed that two different SAGE tags, with an occurrence of 3, were identified for the unannotated deduced protein zgc:103482 (up|Q5XJA5|), due to the presence of a 3'-untranslated region that could be extended by 127 bp, as revealed by the nucleotide sequence of dbEST clone gb|BM101604|. The unique common translated region of both transcripts encoded a protein that was part of the described proteomic profile.

Vitellogenin (VTG) derivatives were identified in most of the gel pieces excised from the 1D- or 2D-PAGE. It should be noted that VTG 1 (*vg1*) SAGE tag (occurrence 4) and VTG 3 (*vg3*) multiple-matched SAGE tag (occurrence 3) were extracted from ZEBRAOV. Other annotated cleaved proteins revealed by the identification of specific peptide sequences with different mass values for the protein of interest were ZP glycoprotein forms, alpha 1 and alpha 8 like 4 tubulins, enolase 3, elongation factor 1-gamma, and nothepsin. Annotated proteins with non-cleavage posttranslational modifications, predicted by variation of the isoelectric point of the protein of interest, were: VTG derivatives, ZP glycoproteins, alpha 1 and alpha 8 like 4 tubulins, enolase 3, elongation factor 1-gamma, mitochondrial aldehyde dehydrogenase 2, chaperonin containing TCP1 subunit 6A, serpin a1, creatine kinase, and pyruvate kinase.

The zebrafish follicle protein repertoire determined by proteomic analysis (Table 5) and the corresponding transcript levels inferred from ZEBRAOV or EST count from ovary libraries ID.9767 and ID.15519 was compared with the protein repertoire deduced from transcripts expressed at over 0.15% in human ovary cDNA libraries ID.4908, ID.5611, and ID.10552 (Table 6). Some of the proteins identified in zebrafish fully-grown follicles have a high, i.e. *bactins, tubb2*, *ldhb*, and *rplp0*, or moderate, i.e. glyceraldehyde-3-phosphate dehydrogenase (*gapd*), ribosomal proteins S3 (*rps3*), elongation factor 1-gamma (*eef1g*), transcript count of homologous UniGene clusters in human ovary cDNA libraries. However, human clusters homologous to ribosomal proteins L7a (*rpl7a*) and SA (*rpsa*), pyruvate kinase (*pkm2*), and enolase 1 alpha (*eno1*) were highly expressed in human cDNA libraries, while the homologous transcripts were counted at very low levels in the zebrafish transcriptome, even if these proteins had been identified by proteomic analysis. It should be noted that ZEBRAOV contained an additional enolase family member homologous to enolase 3 beta (*eno3*), with a moderate expression level (ug|Dr.25678|, 0.109%), while its human annotated counterpart (ug|Hs.224171| was expressed at very low levels or not in human cDNA libraries used for analyses. Enolase transcript (ug|Dm.18435|) was detected in *Drosophila *ovary dbEST libraries ID.1058 (0.052%) and ID.1059 (0.102%).

## Discussion

As in other vertebrates [1], somatic gonadal cells in zebrafish surround a single oocyte to establish a follicle [31]. The entire folliculogenesis process, from primary growth to post-vitellogenic stage takes about ten days in zebrafish [32]. Since large numbers of follicles at different developmental stages are easily obtained year round in this species, zebrafish offer an excellent alternative model for analysing some fundamental aspects of ovarian development and regulation [33], as well as identifying conserved maternal factors [29], which are important in early stages of embryo development. This study analysed the transcriptome and proteome of zebrafish fully-grown ovarian follicles and compared these data with other animal ovary/follicle/egg molecular phenotypes published or available in public sequence databases.

The delineation of the transcriptome of teleost fish ovaries has already been evaluated using large-scale EST sequencing of cDNA libraries [7-9], subtractive hybridisation [11], and microarray-based analyses [12]. These large-scale strategies were used, together with digital differential display analysis, to identify expressed genes specific to ovaries/follicles/oocytes and early embryos in mice [34-39], bovines [40-42], rats [43,44], and humans [45]. While these methods give an idea of transcript abundance or enrichment in a specific tissue, it has been demonstrated that the SAGE method is reproducible [46], provides an unbiased, quantitative report of gene expression, that may be correlated with microarray data [47,48], and seems more efficient than EST-based methods for discovering novel genes and spliced variant transcripts [13]. However, one limitation of the SAGE method is the presence of transcripts that produce multiple matched tags [[49-51], this study]. SAGE has been applied to human oocytes [16,17], and silkworm eggs [52], and successfully identified differentially expressed genes in human ovarian carcinomas and normal ovarian surface epithelium [53]. Large-scale analyses of proteomes from mouse oolemmal proteins [54], matured pig oocyte proteins [55], microtubule-associated proteins from *Xenopus *egg extracts [56], and proteins extracted from *Drosophila *oocytes [57] have also been carried out. To our knowledge, no previous study had analysed the transcriptome and proteome profiles of samples of ovarian origin at the same biological stage on a large-scale.

The transcript repertoire obtained using the SAGE method is an accurate picture of gene expression on both qualitative and quantitative levels and gives a global expression profile of transcripts present in zebrafish fully-grown ovarian follicles. Sequencing of 27,486 SAGE tags identified 11,399 different tag species, classified into 3,437 UniGene clusters with tags in position R1 or R1cr, including 3,329 tag species with an occurrence greater than one. Comparative analysis of transcriptional activity, using the ZEBRAOV SAGE tag database and dbEST libraries currently available for zebrafish ovaries, revealed a globally similar pattern between ZEBRAOV and the ID.9767 library (Figure 2). However, a clearly different quantitative pattern was obtained with library ID.15519, due to an over-representation of the number of EST sequences attached to a small number of unique transcripts. This bias is commonly observed with the EST sequencing method [13]. Consequently, some of the abundant transcripts found by the SAGE method were not detected in library ID.15519. For example, a transcript moderately similar to zygote arrest 1 (ZAR1), with a domain similar to the atypical homeodomain (PHD) finger motif found in ZAR1 in vertebrate species, including zebrafish [58], is highly expressed in ZEBRAOV, moderately in library ID.9767, and not in library ID.15519. In a second example, the transcript of signal sequence receptor beta (*ssr2*), also called translocon-associated protein beta, was detected at high levels with SAGE, and very low levels with dbEST ovary library sequencing (Table 2). This protein is part of the translocon-associated protein (TRAP) complex required for the translocation of nascent polypeptides into the lumen of the endoplasmic reticulum, and the corresponding zebrafish *ssr2 *mRNA is maternally supplied to the egg [59]. We also found the ZEBRAOV database around 1.8 times more sensitive than EST sequencing in detecting transcripts with a characterized maternal genetic contribution (Table 3), even if the number of cDNA clones sequenced to generate the ZEBRAOV SAGE tags database was around 21 times lower. However, this number is not sufficient to identify some of these transcripts, as demonstrated with the maternal-effect *vasa *transcript that was not detected in ZEBRAOV. The *vasa*-like genes are expressed in the germ cells of many animal species [60], including zebrafish oocytes and early-stage embryos [61,62]. Furthermore, the presence of unmatched tags in the SAGE library generated from ovarian follicles indicates the presence of genes in the zebrafish genome or spliced variant transcripts that had not previously been identified by EST data. A broader snapshot of gene expression was therefore obtained by SAGE, as previously reported [13,46]. It should be pointed out that some of these unidentified transcripts are largely expressed in the zebrafish fully-grown follicle transcriptome.

Comparison of the transcriptome of zebrafish fully-grown follicles as evaluated by the SAGE method with ovary/egg transcriptomes available for other animal species revealed both similarities and differences. SAGE revealed the presence of several tags corresponding to novel transcripts, some highly expressed in the zebrafish fully-grown follicle transcriptome and well-conserved in vertebrates. As expected, some of the most abundant transcripts identified in zebrafish, corresponding to some ribosomal proteins or translated to housekeeping genes, including beta-actins, and tubulins, or well known ovary-enriched proteins, like ZP protein isoforms or cyclins [7,63], are well-conserved in the ovarian transcriptomes of other fish species. Homologous highly-expressed transcripts were also recovered in mammals and *Xenopus *transcriptomes and, to a lesser extent, in silkworm, nematode, sea urchin, and amphioxus egg profiles [34,37] (Table 4, and Results section). Some of these transcripts are members of multigene families that may be widely expressed in the zebrafish fully-grown follicle transcriptome, e.g., ZP protein isoform transcripts. This high transcript level may be restricted to a few members of other gene families, as illustrated with claudin genes. Claudins, the major tight junction transmembrane proteins, are members of the tetraspanin protein superfamily that mediate cellular adhesion and migration [64]. Numerous claudin genes have been identified in zebrafish [65] but only the *cldnb *transcript was recovered at very high levels in ZEBRAOV and library ID.9767, and *cldng *in library ID.9767. Some of the claudin isoform transcripts of maternal origin are then downregulated in the early stages of zebrafish embryogenesis [66].

The most abundant transcript in zebrafish fully-grown follicles belongs to a large conserved protein family containing one domain with sequence similarities to the galactose/rhamnose-binding lectin domain found in numerous proteins with sugar binding properties. This domain was initially characterized in sea urchin (*Anthocidaris crassispina*) egg lectin (SUEL) [67]. It was then characterized in rhamnose-binding lectins from of rainbow trout eggs (*Oncorhynchus mykiss*), which consist of two homologous SUEL domains repeated in tandem [68]. It has been suggested that this domain plays a role from egg maturation to fertilization [69]. Rhamnose-binding lectin in catfish (*Parasilurus asotus*) is composed of three tandem-repeat domains homologous to the SUEL lectin domain [70]. A cysteine-rich domain homologous to the SUEL protein has been also identified in the N-terminal part of mammalian latrophilin-2 precursor protein [71].

The SAGE approach also revealed numerous transcripts highly expressed in zebrafish that were not previously known to be significantly expressed by zebrafish ovaries, including *mt2*, *hsp90b*, *ldhb*, *atp5g*, *fth1*, *cirbp, rplp0*, and 40S ribosomal protein S27a. The relative abundance of molecules stored in oocytes may differ between species but some of the abundant transcripts found in zebrafish follicles are common highly-expressed transcripts in vertebrate ovaries/unfertilized eggs (Table 4). It is noteworthy that almost all ribosomal protein transcripts identified from the SAGE tags, expressed at over 0.15%, were recovered below this limit from the two selected zebrafish ovary cDNA libraries. In some cases, these differences may be related to the loss of these small size transcripts after size selection of cDNAs during construction of the libraries, a process that did not occur using the SAGE method.

Short *mt2 *transcript is a very good example of the quantitative as well as qualitative original data obtained after SAGE analysis. We found that *mt2 *was very abundantly expressed in zebrafish oocytes, at a level ten times higher than that previously inferred from analysis of zebrafish ovary dbEST libraries (Table 2). This difference in *mt*2 transcript levels may be due to the loss of this small size transcript during cDNA library construction. An enrichment of this transcript in fully-grown oocytes *versus *ovaries is less plausible due to the asynchronous development of zebrafish ovaries, containing oocytes at different stages in development [31]. In addition, whole-mount *in situ *hybridization demonstrated a strong stage-dependent *mt2 *polarized hybridization signal in the cytoplasm of zebrafish oocytes (Figure 3). These data are consistent with the metallothionein activity content of zebrafish oocytes [72] and the presence of this transcript before the mid-blastula transition of the embryo [73]. Metallothionein transcripts were also recovered from sea urchin egg and salmon ovary dbEST libraries, as well as lizard ovarian follicles (*Podarcis sicula*), with the highest level in ovulated eggs [74]. Expression of the rat *Mt2 *gene is also strongly regulated during primordial follicle assembly and development in rat ovaries [44]. SAGE may also help to distinguish between the expressions of several isoforms at the 3'-end of a transcript. In the same UniGene *mt2 *cluster a second *mt2 *transcript, identified with its *in silico *SAGE tag, contained an identical sequence in the coding region but a long untranslated 3'-part. This long transcript was not expressed in zebrafish fully-grown follicles. It should be noted that differential expression of 3'-end transcript isoforms was easily identified using the SAGE method, as also demonstrated with the *ccnb2 *transcript.

In addition to the *mt2 *short transcript, abundant transcripts of heavy chain ferritins, including *fth1*, related to metal binding, were also detected in ZEBRAOV. This is in accordance with a disproportionately high number of salmon ovary assembled ESTs seen in GO categories related to heavy metal (copper, iron, and zinc) [9] and the presence of ferritin H mRNA in rainbow trout (*Oncorhynchus mykiss*) eggs [75]. Homologous genes to zebrafish *fth1 *are expressed in all vertebrate ovary dbEST libraries available at UniGene, with very high relative levels in salmon, swine, dog, and human libraries. Ferritin-containing inclusions were demonstrated in yolk platelets of schistosome (*Schistosoma mansoni*) [76], a species in which a female-specific yolk ferritin transcript is expressed at high levels in the vitellarium [77]. Ferritin also occurs in amphibian [78,79] and snail [80] eggs. It should be noted that high-level expression of ferritin H-chain mRNA is observed in metastatic human ovarian tumours [81].

The second significant difference in transcript abundance between zebrafish fully-grown follicle transcriptomes as evaluated by SAGE and the profile defined in ovary cDNA libraries concerned the *hsp90β *transcript (Table 2). Extensive molecular characterization, including zebrafish transcripts, and biochemical studies have revealed that vertebrate members of the heat shock protein 90 (HSP90) family play a post-translational regulatory role within the cell by interacting with several important cellular signalling molecules and transcription factors, such as steroid receptors, and modulating their activity [82]. Homologous transcripts are highly expressed in mouse and human ovaries (Table 4) and a strong HSP90 immunoreactivity was demonstrated in rat primordial germ cells [83]. This signal was also detected in both male and female pre-meiotic germ cells. HSP90 was also identified as one of the highly abundant proteins in mature mouse eggs and is strongly associated with the plasma membrane [54]. In addition, *hsp83 *transcript, the *Drosophila *homologue of the mammalian Hsp90 family of regulatory molecular chaperones, is present at high levels through the end of oogenesis and both maternal and zygotic transcripts are spatially restricted during early embryo development [84]. All these data are consistent with the high transcript level of *hsp90β *in zebrafish ovaries, whereas a high number of *hsp90β *cDNA clones was observed in the library generated from testes but not ovaries [7]. The large discrepancy in the relative level of *hsp90β *transcripts observed between the SAGE and cDNA approaches may be related to an enrichment of this transcript in the terminal stages of folliculogenesis.

Other transcripts highly expressed in zebrafish follicles and consistently represented in vertebrate ovarian transcriptomes are transcripts of ATP synthase, H+ transporting, mitochondrial F0 complex, subunit c (ATP5G), cold-inducible RNA-binding protein (CIRBP), and lactate dehydrogenase B4. *atp5g *is highly expressed in fish ovaries and the encoded protein is one of the chains of the nonenzymatic membrane component (F0) of mitochondrial ATPase in mitochondrial membrane. CIRBP apparently plays an essential role in cold-induced suppression of cell proliferation [85]. One of the *Xenopus *CIRBP homologues is a major RNA-binding protein in fully-grown oocytes and may be involved in translational regulation via modulation of oocyte ribosomal function [86]. Lactate dehydrogenase B transcripts are widely distributed in animal ovarian transcriptomes, with high levels found in mice and humans. It has been previously demonstrated that lactate dehydrogenase B mRNA is one of the most abundant transcripts in fully-grown mouse oocytes [87]. Lactate dehydrogenase mRNA appears to be translated efficiently during oocyte growth and then downregulated during maturation and after fertilization [88].

The egg is a transcriptionally inactive cell and, as such, is a storehouse of maternal mRNA and proteins required for fertilization and initiation of zygotic development. However, many of the proteins comprising the animal egg proteome have yet to be identified, as very few large-scale proteome analyses have been performed. As expected, the zebrafish follicle protein repertoire, determined by proteome analysis, identified ribosomal proteins, ZP family protein members, components of the cytoskeleton, chaperonins, heat shock proteins, and VTG derivatives, but also some proteins not previously reported in ovary protein repertoires, e.g. a Sjogren syndrome antigen B homologous protein (Table 5). This RNA-binding protein binds to several small cytoplasmic RNA molecules, known as Y RNAs, and may stabilize these RNAs, preventing degradation [89]. At least eight proteins, out of a total of thirty-eight deduced using the SAGE assigned transcript method and expressed at over 0.15%, were identified by proteome analysis. The identification of abundant mRNAs without the corresponding translated proteins may be due to insufficient proteome delineation and/or the presence of oocyte stage-specific maternal transcripts, stored inside the oocyte cytoplasm and translated during early embryo development. There were several proteins distributed in over one spot position after 1D-, 2D-PAGE separation and MS/MS (Table 5). While some of them, e.g. creatine kinase (CK), were present in closely isoelectric focusing located spots, suggesting the presence of isoforms or posttranslational modifications, the distribution of other spots, e.g. VTG derivatives, indicates a cleavage of precursor proteins with the presence of lower-molecular-weight derivative fragments. CKs play crucial roles in intracellular energy transfer and expression of a CK brain-type isoenzyme during oogenesis has been demonstrated in rodents [90,91]. A homologous transcript was also identified at high levels in amphioxus eggs (ug|Bfl.4313|, 0,119%). *ckb *mRNA is shown to be maternally supplied in zebrafish embryos [92].

Comparison of the zebrafish follicle protein repertoire deduced from SAGE with the protein repertoire isolated after proteomic analysis revealed that some abundant transcripts identified by their SAGE tags, but not previously reported to be present in abundance in fish ovaries, had corresponding proteins. This was the case of lactate dehydrogenase B4 and, to a lesser extent, ribosomal protein large P0 (Tables 2 and 5). Comparison also revealed that *bactin1 *and *bactin2 *transcripts were differentially expressed in zebrafish ovarian follicles, but their protein sequences were not resolved due to the very high sequence conservation of these duplicated gene copies. On the other hand, some ZP family protein members could be discriminated on the protein level, while the same SAGE tags were generated with *zp2.3 *and *zp2.4 *or *zp3a *and *zp3al *transcripts, due to the high sequence similarities of the 3'-end untranslated part of these transcripts.

Oocyte growth, particularly in oviparous species, is characterized by intense deposition of RNAs and proteins, not necessary of the same nature and origin. These maternal factors can be stored for very long periods of time until their use during embryonic development. Comparison of transcriptome and proteome data revealed that transcript levels provide little predictive value with respect to the extend of protein abundance, taking into account the fact that the protein identification approach used detects relatively abundant proteins from the biological extract, while the mRNA abundance evaluated by SAGE tag frequency varied by over two orders of magnitude (Table 5). Transcript profiling provides a measure of RNA abundance, which may be affected not only by transcription levels but also by RNA processing and degradation. Moreover, not all transcripts are translated and RNA abundance may not correspond to protein levels. High transcription and translation rates during folliculogenesis and oocyte growth are followed by differential translational silencing and degradation of many mRNA species, especially at the end of the oocyte growth phase [2,93]. The identification of zebrafish follicle proteins, e.g. pyruvate kinase and enolase I, by proteome analysis, with very low corresponding transcript levels but very high homologous transcript counts in human ovary transcriptomes used as an external reference, suggests a downregulation of the quantity of these transcripts and storage of the proteins at the end of zebrafish folliculogenesis.

A comparison of transcriptome and proteome data revealed two proteins encoded by *chia *and *nots *without corresponding transcripts in ZEBRAOV. *chia *is related to the multiple chitinases genes identified in rainbow trout and Japanese flounder [94], as well as, to a lesser extent, the acidic mammalian chitinase precursor in humans [95]. While the molecular functions of these proteins are related to chitin binding and chitinase activity, the functionality and origin of the protein identified in zebrafish fully-grown follicles remains to be determined. However, the presence of a small amount of *chia *transcript in multiple follicular stage zebrafish cDNA libraries ID.9767, ID.15519 supports a stage-specific transcription of this gene during zebrafish folliculogenesis as previously demonstrated by the downregulation of the transcription of some fish maternal genes, e.g. VTG/very-low density lipoprotein receptor [96] at the end of oogenesis. A high rate of protein deposition has also been observed during oocyte growth in oviparous species via a receptor-mediated endocytosis of exogenous precursors. The presence of an abundant protein in the repertoire without a corresponding transcript in ZEBRAOV may be due to endocytosis of the protein from the plasma to the oocyte. Zebrafish *vg1 *and *vg3 *are mainly expressed in the liver and, to a lesser extent, in several non-liver tissues, including the adipocytes associated with several organs, such as ovaries [97]. This may explain the presence of a limited amount of *vg1 *and *vg3 *transcripts in ZEBRAOV (Table 5). These precursor proteins are synthesized outside oocytes during vitellogenesis, specifically incorporated in the oocyte by receptor-mediated endocytosis and cleaved into yolk proteins. The identification of nothepsin in zebrafish fully-grown follicles by proteome analysis although no transcript was detected in the ovary by Northern blot [98], EST sequencing of ovary cDNA libraries, or SAGE (this study), strongly suggests an extraovarian origin for this enzyme that may be present in the plasma of females undergoing vitellogenesis. Zebrafish *nots *encodes a paralogous aspartic proteinase related to endoproteolytic proteinases, such as cathepsin D, cathepsin E, and pepsin. This gene is specifically expressed in the liver under estrogenic control [99]. The sexual dimorphic expression of *nots *may be related to the reproductive process, like VTG precursor processing, or other sex-specific proteins inside the oocyte cytoplasm.

## Conclusion

This study provides a complete sequence data set of maternal mRNA stored in zebrafish germ cells at the end of oogenesis. This catalogue contains highly-expressed transcripts that were not previously known to be significantly expressed in the fish ovaries, including some that are part of a vertebrate ovarian expressed gene signature. Comparison of transcriptome and proteome data identified downregulated transcripts or proteins potentially incorporated in the oocyte by endocytosis. The molecular phenotype described provides groundwork for future experimental approaches aimed at identifying functionally important maternal transcripts and proteins involved in oogenesis and early stages of embryo development.

## Methods

### Isolation of fully-grown ovarian follicles

Zebrafish, *Danio rerio*, were obtained from our facilities and maintained at 28.5°C on a 12L:12D photoperiod. Salts (0.23 g/l Instant Ocean, Aquarium System, Inc, Mentor, USA and 0.1 g/l CaSO_4_, 2H_2_O) were added to reverse osmosis (Optima 60, Veolia Water STI, Blagnac, France) purified water in order to ensure an optimal water quality. The zebrafish ovaries undergo asynchronous development and oocyte development is divided into five stages: I (primary growth), II (cortical alveolus or pre-vitellogenic), III (vitellogenic), IV (maturation), and V (mature egg) [31]. Sexually mature females were anaesthetized by immersion in 2-phenoxyethanol (1/2000, v/v) and fully-grown follicles (diameter > 0.69 mm) [31] were obtained by gently squeezing the abdomen. Histological analysis demonstrated that, in some areas, the stripped oocytes were covered with a single layer of granulosa cells, attached to the zona radiata (data not shown).

### SAGE library construction

Total RNAs were isolated from about 250 fully-grown follicles, isolated from five none in-bred mature females, using the NucleoSpin RNAII kit (Macherey-Nagel, Duren, Germany). Fifty micrograms of total RNAs were used to generate the SAGE library using the I-SAGE kit (Invitrogen, Cergy Pontoise, France). The SAGE library was constructed according to the manufacturer's instructions, with minor modifications. Briefly, total RNAs were bound to magnetic Dynal oligo(dT) beads and cDNAs were synthesized directly on oligo(dT) beads. cDNAs were digested with *Nla*III at 37°C for 1 h and divided into two equal parts, pools A and B. These pools were ligated at 16°C for 2 h to specific adapters, adapX (5'-TTTGGATTTGCTGGTGCAGTACAACTAGGCTTAATAGGGACATG-3') and adapY (5'-TTTCTGCTCGAATTCAAGCTTCTAACGATGTACGGGGACATG-3'), containing the priming sites for PCR amplification at the 5'-end and the type IIS restriction endonuclease *Bsm*FI site at the 3'-end. The 3'-ends of the adapters were modified with an amino group to prevent self-ligation. The two ligation products were then cleaved with the tagging enzyme *Bsm*FI at 65°C for 1 h. The resulting tags from pools A and B were ligated at 16°C overnight in a 3 μl mixture to form ditag cassettes. The ligated ditag mixture was diluted 1:110 (v/v), and 1 μl was used in a 50 μl PCR mixture. A total of 300 ditag PCR amplifications were performed for 33 cycles using the following primers derived from adapX (5'GGATTTGCTGGTGCAGTACA-3') and adapY (5'-CTGCTCGAATTCAAGCTTCT-3'), respectively. Individual ditag PCR products (100 bp) were purified on 12% (w/v) polyacrylamide gel. Adapters (40 bp) were removed from ditags by *Nla*III digestion at 37°C for 2 h 30 min and ditags without adapters (26 bp) were purified on 12% (w/v) polyacrylamide gel. Purified ditags (26 bp) were ligated together at 16°C overnight and resolved in an 8% (w/v) polyacrylamide gel. Concatemer fractions ranging from 0.3 to 0.6 kb, 0.6 to 1.5 kb, and over 1.5 kb, were purified separately. The purified concatemers were cloned into the *Sph*I site of the pZErO-1 plasmid (Invitrogen, Cergy Pontoise, France). The ligated mixture was transformed into One Shot TOP10 electrocompetent cells (Invitrogen, Cergy Pontoise, France). Positive transformants were selected by plating on low-salt Luria-Bertani plates containing 50 μg/ml Zeocin and incubating at 37°C for 24 h. The concatemer sizes were screened for 10% of total clones by colony PCR using Sp6 forward and T7 reverse primers.

### SAGE library sequencing

High throughput sequencing reactions were carried out by Genome Express (Meylan, France). Automatic tag detection, extraction, counting and quality control were undertaken by Skuld-Tech (Montpellier, France). Ditags longer than 50 bp (2.97%), fewer than 24 bp (0.4%), or repeated ditags (2.69%) were discarded and not taken into account for tag number calculation. Contamination rates with linker and ribosomal 18 S and 28 S RNA sequences were 0.2% and 0.22%, respectively. The ZEBRAOV was generated from 576 sequenced clones containing inserts originating from the 0.6 to 1.5 kb concatemer fraction. Consequently, 96% of the sequenced clones have concatemers with over 500 bp, resulting in an average SAGE tag number of 47 per concatemer.

### Data analysis and tag-to-gene mapping

To our knowledge, we were the first to carry out SAGE analysis on a fish species. Consequently, linking an experimental SAGE tag to an annotated transcript required the development of a species-specific SAGE tag database from the tags extracted from ESTs or transcripts available for a particular fish species. FISHTAG, a generic computer package written in PERL and implemented on a UNIX workstation, was designed for automatic extraction and annotation of SAGE tags from the sequences deposited in UniGene and TIGR public sequence databases (Rousselot *et al*., in preparation). Briefly, three 14-long nucleotide *in silico *tags, identified with the 5'-CATG ending sequence, were extracted from each reference transcript sequence at the first three sense positions, starting from the 3'-end of the transcript, and named R1, R2, or R3 in the presence of a polyadenylation signal and poly(A) tail. The tags were named Rn1, Rn2, Rn3 for EST sequences without a polyadenylation signal, a poly(A) tail, or both. Furthermore, the corresponding *in silico *tags of complementary reversed antisense sequences of deposited ESTs called "3'-reads" were also extracted and named R1cr, R2cr, or R3cr in the presence of a polyadenylation signal and poly(A) tail. The tags were named Rn1cr, Rn2cr, Rn3cr for EST sequences without a polyadenylation signal, a poly(A) tail or both. A table was constructed and additional data, like EST cluster descriptions, GenBank™ annotations, and URL links to the UniGene or TIGR sites were attached to each extracted *in silico *tag. This table was then used as a reference for tag-to-gene mapping by comparison between the experimental SAGE tags and the EST-derived *in silico *extracted SAGE tags.

The zebrafish sequence data were downloaded from UniGene and TIGR ftp sites [100,101]. There were 673,076 public UniGene sequence entries (Build#87), including 99,968 3'-read entries, in 31,681 UniGene clusters. Data retrieved from TIGR contained 33,752 unique TC leader sequences (release 16.0). The EST sequences used and included in UniGene were from more than two-hundred dbEST libraries originating from at least twenty different zebrafish tissues or developmental stages [102]. The *in silico *zebrafish SAGE-tag database, ZEBRATAG, generated using FISHTAG, was used to combine the copy number of each experimental SAGE tag from the ZEBRAOV library with its annotation.

### Ovary/egg cDNA library sequence data used

The teleost fish ovary dbEST cDNA libraries currently available at the GenBank™ database and used to analyse their EST sequences were: ID.15519 (NIH_ZGC_5) with 13,029 EST sequences classified into 2,610 clusters and ID.9767 (Gong zebrafish ovary), with 11,344 EST sequences classified into 2,794 clusters, for zebrafish (*Danio rerio*) (UniGene Build#87), ID.15587 (AGENAE rainbow trout normalized ovarian library) with 4,936 EST sequences classified into 274 clusters for rainbow trout (*Oncorhynchus mykiss*) (UniGene Build#16), ID.11967 (Fugu UT13 adult ovary) with 2,545 EST sequences classified into 705 clusters for Fugu (*Takifugu rubripes*) (UniGene Build#13), and ID.15459 (Atlantic salmon ovary cDNA library) with 2,269 EST sequences classified into 849 clusters for salmon (*Salmo salar*) (UniGene Build#8).

The other vertebrate dbEST cDNA library accession numbers used for comparison were ID.10552 (NIH_MGC_109), with 10,652 sequences classified into 2,973 UniGene entries, ID.4908 (NIH_MGC_9), with 12,849 sequences classified into 3,289 UniGene entries, and ID.5611 (NIH_MGC_66) with 8,540 sequences classified into 3,060 UniGene entries for human (*Homo sapiens*) ovaries (UniGene Build#187), ID.15096 (full-length enriched swine cDNA library, adult ovary) with 26,127 sequences classified into 4,770 UniGene entries for swine (*Sus scrofa*) (UniGene Build#34), ID.10029 (NIA mouse unfertilized egg cDNA library, long) with 13,201 sequences classified into 1,939 UniGene entries, ID.1389 (mouse unfertilized egg cDNA library) with 3,098 sequences classified into 1,228 UniGene entries, and ID.14142 (NIA mouse unfertilized egg cDNA library, long 1) with 6,443 sequences classified into 1,821 UniGene entries for mouse (*Mus musculus*) oocytes (UniGene Build#148), and ID.9909 (XGC-egg) with 65,236 sequences classified into 7,968 UniGene entries for *Xenopus tropicalis *(UniGene Build#26).

The other non-vertebrate dbEST cDNA library accession numbers used for comparison were ID.13749, with 6,003 EST sequences classified into 1,775 clusters for unfertilised sea urchin (*Strongylocentrotus purpuratus*) eggs (UniGene Build#9), ID.17404, with 38,522 EST sequences classified into 3,720 clusters for amphioxus (*Branchiostoma floridae*) eggs (UniGene Build#1), and ID.1977, with 53,273 EST sequences classified into 5,777 clusters for fertilized nematode (*Caenorhabditis elegans*) eggs (UniGene Build#25). In addition, a silkworm (*Bombyx mori*) egg SAGE tag library [52] was also used.

### Whole-mount *in situ *hybridization

Follicles were fixed in 4% paraformaldehyde overnight at 4°C, rinsed three times in phosphate buffered saline (PBS) buffer, twice in methanol, and stored at -20°C in methanol until used. A first primer pair (sense oligonucleotide 5'-CAGCAGAATCATGCGCTC-3' and antisense oligonucleotide 5'-CAACATATGGACGACAGG-3') was used to amplify a 611 bp of a cDNA fragment from nucleotides -10 to +601 (numbered from the translation initiator codon) of a transcript (GenBank™/EBI (gb) Data Bank accession number gb|BM141044|) with similarities to human latrophilin-2, identified as a rhamnose-binding lectin. A second primer pair (sense oligonucleotide 5'-GACTGGAACTTGCAACTG-3' and antisense oligonucleotide 5'-GACGGTACAGGAAACAGAT-3') was used to amplify a 445 bp cDNA fragment from nucleotides +24 to +468 of *mt2 *short transcript (gb|BC049475|). A third primer pair (sense oligonucleotide 5'-TATCCAGAAGCATCGTCAGG-3' and antisense oligonucleotide 5'-CCTTCACATCACACTCATGC-3') was used to amplify a 764 bp cDNA fragment from nucleotides +57 to +820 of *dazl *transcript (gb|NM_131524|). A fourth primer pair (sense oligonucleotide 5'-AGGCTGCTTCAGGAGACC-3' and antisense oligonucleotide 5'-CCTAAAGAAGTGACGGTACC-3') was used to amplify a 769 bp cDNA fragment from nucleotides +550 to +1318 of *ccnb1 *transcript (gb|AB040435|). These amplified cDNA fragments were used as templates to generate the corresponding RNA probes. Both antisense- and sense-digoxigenin- and fluorescein-labelled RNA probes were obtained using T7 or SP6 RNA polymerase (Promega, France) and the digoxigenin/fluoresceine RNA labelling mix (Roche, Germany), following the manufacturer's instructions. RNA probes were purified using the ProbeQuant G-50 micro columns (Amersham Biosciences, England) and checked for purity by denaturing agarose gel electrophoresis. Whole-mount *in situ *hybridization was carried out as previously described , with minor modifications. The PBS buffer used contained 0.04% (w/v) KCl. Samples were treated with 20 μg/ml proteinase K for 20 min and hybridized at 58°C with 50% formamide. Two-colour whole-mount *in situ *hybridization was carried out with a fluorescein-labelled RNA probe to detect the most-strongly expressed gene, then with the digoxigenin-labelled RNA probe. Samples were mounted in 100% glycerol and observed under an Eclipse E1000 Nikon microscope. Histology was performed according to the procedure previously described [103].

### Protein extraction

Proteins were extracted from zebrafish fully-grown follicles using TRI Reagent™ (Sigma-Aldrich, U.S.A.) [104]. A 100 μl aliquot of frozen follicles in L-15 was mixed with 1 ml of cold TRI Reagent and incubated at room temperature for 10 min. 200 μl chloroform were added and mixed, incubated at room temperature for 10 min and centrifuged at 10,000 g at 4°C for 10 min. The protein phase was separated from the DNA and RNA and the proteins precipitated with 300 μl 100% ethanol at room temperature for 10 min. After centrifugation at 2,000 g at 4°C for 10 min, the supernatant containing the proteins was precipitated with isopropanol at room temperature for 10 minutes, and then centrifuged at 10,000 g at 4°C for 10 min. The protein pellet was washed twice with 2 ml 0.3 M guanidine hydrochloride in 95% ethanol and incubated for 20 min at room temperature, then centrifuged at 6,500 g, at 4°C for 10 min. The pellet was further washed three times with 10 ml 100% ethanol for 20 min at room temperature and centrifuged at 6,500 g, in 4°C for 10 min. The remaining salts were removed by adding 1.5 ml 80% cold acetone (-20°C) and centrifuging at 6,000 g at 4°C for 10 min three times. The air-dried pellet was used for 1D- and 2D-PAGE.

### 1D-, 2D-PAGE and tandem mass spectrometry

Protein pellets were dissolved in 2D-PAGE sample buffer containing 7 M urea, 2 M thiourea, 2% CHAPS, 65 mM DTT, 1.25% ampholytes (pH 3–10, BioRad) with a trace of bromophenol blue. The protein amounts were assayed with the Bradford reagent (BioRad, Hercules, U.S.A.). For isoelectric focusing, 600 μg of each protein sample were applied to 11 cm IPG Strips (pH 3–10 NL, BioRad). Isoelectric focusing was at 70,000 Volt/hr with an IPG-Phor System (Amersham Biosciences), followed by second-dimension electrophoresis, using pre-cast Criterion Tris-HCl gels (4–20% Linear Gradient, BioRad). 1D SDS-PAGE (133 × 87 × 1 mm) was performed on a home-made polyacrylamide gradient gel 8–16%. The gels were stained with See-Band Forte (GeBA). Images were taken with a FlourS imager (BioRad) and the images were analysed using PDQuest software (BioRad). Spots were excised, in-gel digested with trypsin, and identified either by peptide mass fingerprinting and CID using a 4700 MALDI-TOF-TOF mass spectrometer (Applied Biosystems) or a nano-capillary RP-HPLC and ESI-QIT mass spectrometer (LCQ-Deca, ThermoFinnigan). The MS data was analysed using Sequest [105], Pep-Miner [106] and Mascot [107] software tools and searching the NCBInr or ZFIN Zebrafish databases. Each peptide identified was then manually checked against the corresponding Swiss-Prot/TrEMBL/PIR UniProt Data Bank entry at the UniProt web site [108]. The presence of a protein in zebrafish fully-grown follicle protein extract was confirmed when at least one peptide sequence perfectly matching the target UniProt protein entry appeared in the proteome database at least twice.

## Authors' contributions

A.K-G., carried out the genomics and bioinformatics studies, A.M. participated in the genomics as well as the molecular biology experiments, T.G. carried out the proteomics studies, J.F. performed the *in situ *hybridization experiments, A.A. participated in the proteomic analysis, and P.J.B. carried out the computational analyses, coordinated the work, and drafted the manuscript. All authors read and approved the final manuscript.

## Figures and Tables

**Figure 4:**
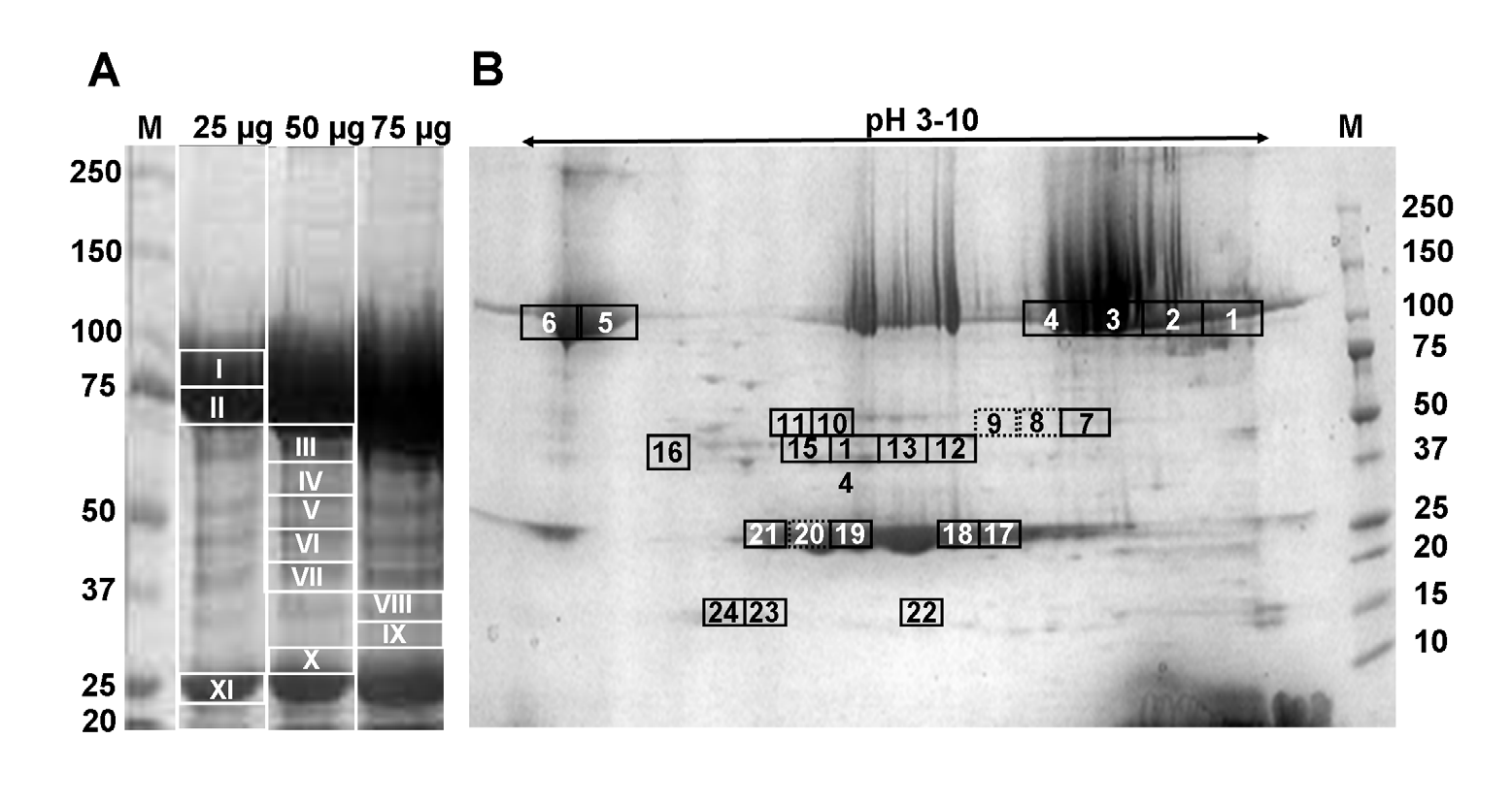
**Electrophoretic pattern of zebrafish fully-grown follicle proteins**. *A*, SDS-polyacrylamide gradient 8–16% slab gel of total yolk proteins with increasing amounts of proteins deposited per lane. Eleven gel slices, numbered I to XI, were cut after staining the gel with See-Band Forte (GeBA). *B*, Two-dimensional polyacrylamide gel electrophoresis of total yolk proteins. Twenty-four spot areas, numbered 1 to 24, were excised after staining the gel with GeBA. In both procedures, gel pieces were in-gel digested with trypsin and the resulting peptides identified by mass spectrometry (see Table 5). The rectangles from which VTG derivatives were isolated are identified with a solid lined line while the rectangles with no derivatives (numbers 8, 9, and 20) are labelled with a broken line. M, relative molecular weights of the standards × 10^-3^.

**Table 1:** Transcripts expressed at over 0.15% in zebrafish fully-grown ovarian follicle as evaluated by the presence of their corresponding SAGE tag

**Tag N°**	**SAGE-tag**	**Nbr**	**Rank**	**GenBank**	**Cluster**	**Gene**	**Description**
1	CATGAAATAAACGC	284	R1	BM141044	Dr.12439		Transcribed locus similar to rhamnose-binding lectin
2	CATGTAAGACATCC	226	R1	BC045879	Dr.1109	*bactin2*	Beta-actin2
3	CATGCAGTGCTTTT	209	R1	BM531116	Dr.46793	*h2b*	Transcribed locus similar to H2B histone family
4	CATGAAAAAAAAAA	153	[146]				
5	CATGTCCTTTGTCT	142	R1	BC049475	Dr.47289	*mt2*	Metallothionein 2
6	CATGTGAACTGTTT	139	[7]				
7	CATGACCGTTTTTA	130	R1	BC078260	Dr.26975	*cldnd*	Claudin d
8	CATGCAAAGGAAAC	124	R1	BC055519	Dr.47296		Zgc:66160
9	CATGCTAGCCTAAT	122	R1cr	BM861564	Dr.5628		Similar to contains element MSR1 repetitive element/ZP1-related
10	CATGGTGAAACCTG	119	R1	AF068772	Dr.31066	*hsp90b*	Heat shock protein 90-beta
11	CATGTTGATGAGGG	113	R1	BC044190	Dr.729	*ldhb*	Lactate dehydrogenase B4
12	CATGTAAAACCAAA	108	R1	AF057040	Dr.25213	*bactin1*	Beta-actin1
13	CATGTATTTCACTA	98	R1	BC045894	Dr.1043	*atp5g*	ATP synthase, H+ trans., mit. F0 complex, subunit c (subunit 9)
14	CATGGATATTTAAC	94	R1	BI979482	Dr.47212		Transcribed locus, moderately similar to thymosin beta 10
15	CATGCATTTTCTAA	90	R1cr	BM571854	Dr.19916	*zp2.3*	Egg envelope protein ZP2 variant B [zp2.3]
15	CATGCATTTTCTAA	90	R1	BC093133	Dr.23439	*zp2.4*	Zp2.4 protein
16	CATGACAACTAAAA	89	R1	BC071411	Dr.989		Zgc:56419 similar to stress-associated endo. reticulum protein 1
17	CATGGTGTGATGGA	82	[5]				
18	CATGGAAAAAAAAA	80	[38]				
19	CATGAGAAACGTTT	77	R1	BC067692	Dr.26977	*zp3b*	Zona pellucida glycoprotein 3b
20	CATGGTGGTGTTGA	75	R1	NM_131331	Dr.30322	*zp3*	Zona pellucida glycoprotein 3
21	CATGCAATCATATT	75	R1	BM533765	Dr.13590		Transcribed locus, moderately similar to zygote arrest 1
22	CATGCAGAAAAATA	73	[5]				
23	CATGGAAGTTGCTT	72	[6]				
24	CATGCAAATGTTTT	71	R1	CK699865	Dr.29860		Transcribed locus
25	CATGAAGTTACTGT	70	[3]				
26	CATGCAGTACTGCG	66	R1	AY561514	Dr.30264	*rpl3*	Ribosomal protein L3
27	CATGCCACTCTTTT	66	[2]				
28	CATGCCATAAGTGC	66	R1	BC054596	Dr.1012		Retinol dehydrogenase 10
29	CATGATATTAATAA	65	[3]				
30	CATGATTTTTTGAA	65	[5]				
31	CATGTAAAGTCGCC	65	R1	BM778414	Dr.1428	*rps15a*	Ribosomal protein S15a
32	CATGGTCTACACGT	64	R1cr	BI325627	Dr.7383		Zgc:73301 similar to cytochrome oxidase III
33	CATGTAAATTTGAA	64	[4]				
34	CATGCTTGTTGGAA	62	R1	BQ260230	Dr.29755		Wu:fe38a04 similar to RL31_HUMAN 60S ribosomal protein L31
35	CATGGGATTCGGAC	61	R1	BM102431	Dr.23788	*gstp1*	Glutathione S-transferase pi
36	CATGGGGTGCTTTT	61	R1	CO351922	Dr.14015		Im:7140333 weakly similar to latrophilin; lectomedin-1
37	CATGACATCACATT	60	[9]				
38	CATGTGCTCCATAC	59	R1cr	BC095579	Dr.5555	*ccna1*	Cyclin A1
39	CATGGCATTTTTAG	58	n.m.				
40	CATGAGGCTGTCTG	55	R1	BC055524	Dr.24903		Zgc:66168 similar to ubiquitin; 40S ribosomal protein S27a
41	CATGTGTTCTGTAT	55	R1	BC095694	Dr.5890		Zgc:112226 similar to cathepsin S
42	CATGTCGATGATGG	54	R1cr	BM103997	Dr.26808	*rpl10a*	Ribosomal protein L10a
43	CATGTTAATAAAAG	53	R1	NM_198809	Dr.5605	*tubb2*	Tubulin beta 2
44	CATGAGGAAAGCTG	52	R1	BC071384	Dr.1335	*rpl36*	Ribosomal protein L36
45	CATGGGCTTCGGTC	52	[4]				
46	CATGTGCTGCTTGT	51	R1	BC045278	Dr.6496	*fth1*	Ferritin, heavy polypeptide 1
47	CATGATCAATGTGA	50	R1	BC075915	Dr.1209	*ssr2*	Signal sequence receptor, beta
48	CATGTGAGGGCTCT	49	[6]				
49	CATGATATGTATTT	48	[3]				
50	CATGAATTGGAGGG	47	n.m.				
51	CATGTCTGTTTAAC	47	R1	BC049058	Dr.11308	*rplp0*	Ribosomal protein, large, P0
52	CATGAAGCCCATTA	46	R1	BC048027	Dr.4947	*cirbp*	Cold inducible RNA binding protein
53	CATGGTTCCTTGGC	46	R1	BQ617300	Dr.1334	*rps25*	Ribosomal protein S25
54	CATGTATCAGTTAT	46	[3]				
55	CATGTTAGTGTGAC	46	[6]				
56	CATGTGAGCCAAAT	45	[3]				
57	CATGTTGTTTTTGT	43	[5]				
58	CATGTAAATGAGAT	42	R1	BC054688	Dr.12480		Clone IMAGE:5604190, weakly similar to Cd27 binding protein

Nbr, is the number of times a tag was counted in ZEBRAOV; Rank, is tag position at sense (R1) or antisense (R1cr) corresponding mRNA sequence; The number in brackets indicates the number of corresponding UniGene clusters in case of a tag matching with more than one transcript, i.e. a multiple matched tag; n.m., no matched tag; Cluster, cluster accession number of zebrafish UniGene database Build#87.

**Table 2:** The most abundant annotated SAGE tags in zebrafish fully-grown follicles and their corresponding UniGene cluster occurrences in two selected zebrafish ovary cDNA libraries

**Tag N°**	Cluster*	**Description**	**SAGE %**	**ID.9767 %**	**ID.15519 %**
**A. Occurrences > 0.15% in ZEBRAOV and cDNA libraries ID.9767 and ID.15519**					
2	Dr.1109	Beta-actin2	**0.822**	**0.317**	**0.752**
9	Dr.5628	Similar to contains element MSR1 repetitive element/ZP1-related	**0.444**	**0.220**	**2.517**
12	Dr.25213	Beta-actin1	**0.393**	**0.326**	**0.698**
15	Dr.19916	Egg envelope protein ZP2 variant B [zp2.3]	**0.327**	**0.397**	**2.610**
15	Dr.23439	Zp2.4 protein	**0.327**	**0.740**	**11.367**
19	Dr.26977	Zona pellucida glycoprotein 3b	**0.280**	**0.264**	**0.399**
20	Dr.30322	Zona pellucida glycoprotein 3	**0.275**	**0.705**	**4.444**
43	Dr.5605	Tubulin beta 2	**0.193**	**0.290**	**0.852**
**B. Occurrences > 0.15% in ZEBRAOV and cDNA library ID.9767**					
1	Dr.12439	Transcribed locus similar to rhamnose-binding lectin	**1.033**	**2.530**	0.046
3	Dr.46793	Transcribed locus similar to H2B histone family	**0.760**	**0.652**	0.008
7	Dr.26975	Claudin d	**0.473**	**0.264**	0.023
8	Dr.47296	Zgc:66160	**0.451**	**0.185**	0.015
14	Dr.47212	Transcribed locus, moderately similar to thymosin beta 10	**0.342**	**0.485**	0.015
41	Dr.5890	Zgc:112226 similar to cathepsin S	**0.200**	**0.194**	0.046
59	Dr.12480	Clone IMAGE:5604190, weakly similar to Cd27 binding protein	**0.153**	**0.300**	0.008
**C. Occurrences > 0.15% in ZEBRAOV and cDNA library ID.15519**					
26	Dr.30264	Ribosomal protein L3	**0.240**	0.053	**0.169**
28	Dr.1012	Retinol dehydrogenase 10	**0.240**	0.062	**0.437**
38	Dr.5555	Cyclin A1	**0.215**	0.123	**0.706**
**D. Occurrences > 0.15% in ZEBRAOV**					
5	Dr.47289	Metallothionein 2	**0.517**	0.053	0.031
10	Dr.31066	Heat shock protein 90-beta	**0.433**	0.079	0.031
11	Dr.729	Lactate dehydrogenase B4	**0.411**	0.035	0.046
13	Dr.1043	ATP synthase, H+ transporting, mit. F0 complex, subunit 9	**0.357**	0.149	0
16	Dr.989	Zgc:56419 similar to stress-associated endo. reticulum protein 1	**0.324**	0.079	0.008
21	Dr.13590	Transcribed locus, moderately similar to zygote arrest 1	**0.273**	0.106	0
24	Dr.29860	Transcribed locus	**0.258**	0.053	0
31	Dr.1428	Ribosomal protein S15a	**0.236**	0.044	0
32	Dr.7383	Zgc:73301 similar to cytochrome oxidase III	**0.233**	0.018	0
34	Dr.29755	Wu:fe38a04 similar to RL31_HUMAN 60S ribosomal protein L31	**0.226**	0.018	0
35	Dr.23788	Glutathione S-transferase pi	**0.222**	0.053	0
36	Dr.14015	Im:7140333 weakly similar to latrophilin; lectomedin-1	**0.222**	0.106	0.015
40	Dr.24903	Zgc:66168 similar to ubiquitin; 40S ribosomal protein S27a	**0.200**	0.088	0.015
42	Dr.31131	Zgc:109948 similar to ribosomal protein L10a	**0.196**	0.062	0.008
44	Dr.1335	Ribosomal protein L36	**0.189**	0.062	0
46	Dr.6496	Ferritin, heavy polypeptide 1	**0.186**	0.123	0.015
47	Dr.1209	Signal sequence receptor, beta	**0.182**	0.009	0.015
51	Dr.11308	Ribosomal protein, large, P0	**0.171**	0.079	0.023
52	Dr.4947	Cold inducible RNA binding protein	**0.167**	0.088	0.100
53	Dr.1334	Ribosomal protein S25	**0.167**	0.035	0

*Zebrafish UniGene Build#87. The values indicated are the percentages of UniGene clusters as evaluated by the percentages of corresponding tags or EST counts in the mRNA population identified in ZEBRAOV SAGE tag database and dbEST libraries. Percentages at over 0.15% were bolded.

**Table 3:** Recovery of selected genes with a characterized maternal genetic contribution in ZEBRAOV and zebrafish cDNA libraries ID.9767 and ID.15519*

**Gene**	GenBank**	**SAGE tag**	**Nbr**	Cluster***	**% SAGE**	**% ID. 9767**	**% ID. 15519**
*ccnb1 *(cyclin B1)	NM_131513	CATGGGTTGAGCAA	25	Dr.25226	0.091	0.229	1.589
*pou5f1 *(POU domain, class 5, trf 1)	NM_131112	CATGCTCACCCGCA	2	Dr.258	0.007	0.035	0.008
*catnb *(Beta-catenin 2)	AF329680	CATGCAGAATAAAG	13	Dr.28281	0.047	0.018	n.d.
*cdc25 *(cdc25)	CO352749	CATGTGTGTGTTCA	6	Dr.8228	0.022	0.035	n.d.
*smad5 *(MAD homolog 5)	AF127920	CATGTATCTCTACT	1	Dr.20558	0.004	0.027	n.d.
*zorba *(Orb/CPEB-related)	NM_131427	CATGCAGAGCTATT	14	Dr.614	0.051	n.d.	0.038
*dazl *(daz-like gene)	NM_131524	CATGGGAGTTGTGG	2	Dr.8159	0.007	n.d.	0.015
*oep *(one-eyed pinhead)	NM_131092	CATGTCATCTTAAC	1	Dr.581	0.004	n.d.	0.008
*foxh1 *(forkhead box H1)	NM_131502	CATGAGAATGCAGT	1	Dr.7743	0.004	n.d.	0.008
*acvr2b *(activin receptor Iib)	AF069500	CATGCAAGACTGTG	2	Dr.21047	0.007	n.d.	n.d.
*pbx4 *(pre-B-cell leukemia trf 4	NM_131447	CATGTAGGGTGTGT	1	Dr.4926	0.004	n.d.	n.d.
*snai1a *(snail homolog 1a)	NM_131066	CATGCTTTTTCTTT	1	Dr.15	0.004	n.d.	n.d.
*cth1 *(cth1)	NM_130939	CATGTTTAACCCTG	n.d.	Dr.621	n.d.	0.044	0.092
*alk8 *(activin receptor-like kinase 8)	NM_131345	CATGTTTGTGTTGA	n.d.	Dr.606	n.d.	0.018	n.d.
*szl *(sizzled)	NM_181663	CATGCGCTAGTCAG	n.d.	Dr.25497	n.d.	n.d.	n.d.
*cugbp *(bruno-like)	BC076238	CATGAGGAGGACGG	n.d.	Dr.16766	n.d.	n.d.	n.d.
*gsc *(goosecoid)	NM_131017	CATGTACGATGGCA	n.d.	Dr.289	n.d.	n.d.	n.d.
*sox19 *(SRY-box containing gene 19)	BC078221	CATGAGGTTCAAC	n.d.	Dr.20910	n.d.	n.d.	n.d.
*stat3 *(signal trans. & act.trans. 3)	NM_131479	CATGTATATAGTGA	n.d.	Dr.8150	n.d.	n.d.	n.d.
*vasa *(vasa homolog)	NM_131057	CATGGCACAACAGG	n.d.	Dr.559	n.d.	n.d.	n.d.
*notch1a *(notch homolog 1a)	NM_131441	CATGGCTCCGCCTA	n.d.	Dr.11847	n.d.	n.d.	n.d.
*notch1b *(notch homolog 1b)	U57973	CATGAAATGAAACT	n.d.	Dr.11846	n.d.	n.d.	n.d.
*notch2 *(notch homolog 2)	AF334944	CATGTTCACCAAAC	n.d.	Dr.604	n.d.	n.d.	n.d.
*notch3 *(notch homolog 3)	NM_131549	CATGTGCCCAATGA	n.d.	Dr.17815	n.d.	n.d.	n.d.

*The total number of cDNA clones sequenced was 576 to generate ZEBRAOV instead of 11,344 with cDNA library ID.9767 and 13,029 with cDNA library ID.15519. **GenBank accession number of the sequence from which the SAGE-tag was extracted. ***UniGene Build#87. All the tags were at R1 sense position with the exception of *notch1a *and *notch1b *transcripts where a Rn1 sense position was found. Nbr, is the number of times a tag was counted in ZEBRAOV. n.d., the corresponding transcript was non-detected. The values indicated are the percentages of UniGene clusters as evaluated by the percentages of corresponding tags or EST counts in the mRNA population identified in ZEBRAOV SAGE tag database and dbEST libraries.

**Table 4:** The most abundant annotated SAGE-tags of zebrafish fully-grown follicles and the presence of homologous UniGene clusters in vertebrate ovary/unfertilized egg cDNA libraries

**Tag N°**	Cluster*	**Description**	**Trout**	**Salmon**	**Fugu**	**Xenopus**	**Swine**	**Mouse**	**Human**
**A. Occurrences > 0.15% in ZEBRAOV and cDNA libraries ID.9767 and ID.15519**									
2	Dr.1109	Beta-actin2	**Y**	N	Y*	**Y**	**Y**	**Y**	Y*
12	Dr.25213	Beta-actin1							
9	Dr.5628	Similar to contains element MSR1 repetitive element/ZP1-related	N	N	N	**Y**	N	N	**Y**
15	Dr.19916	Egg envelope protein ZP2 variant B [zp2.3]	N	N	N	**Y**	N	N	**Y**
15	Dr.23439	Zp2.4 protein	**Y**	N	N	n.i.	n.i.	N	N
19	Dr.26977	Zona pellucida glycoprotein 3b	Y*	Y*	N	**Y**	N	N	**Y**
20	Dr.30322	Zona pellucida glycoprotein 3							
43	Dr.5605	Tubulin beta 2	N	**Y**	n.i.	**Y**	Y*	Y*	Y*
**B. Occurrences > 0.15% in ZEBRAOV and cDNA library ID.9767**									
1	Dr.12439	Transcribed locus similar to rhamnose-binding lectin/Latrophilin-2	Y*	Y*	n.i.	**Y**	N	N	**Y**
3	Dr.46793	Transcribed locus similar to H2B histone family	n.i.	n.i.	n.i.	N	**Y**	N	**Y**
7	Dr.26975	Claudin d	N	n.i.	n.i.	Y*	**Y**	N	**Y**
8	Dr.47296	Zgc:66160	N	n.i.	n.i.	**Y**	N	**Y**	N
14	Dr.47212	Transcribed locus, moderately similar to thymosin beta 10	N	n.i.	n.i.	**Y**	**Y**	N	**Y**
41	Dr.5890	Zgc:112226 similar to cathepsin S	N	N	n.i.	N	N	N	N
59	Dr.12480	Clone IMAGE:5604190, weakly similar to Cd27 binding protein	N	n.i.	n.i.	**Y**	N	N	**Y**
**C. Occurrences > 0.15% in ZEBRAOV and cDNA library ID.15519**									
26	Dr.30264	Ribosomal protein L3	N	N	n.i.	N	N	N	Y*
28	Dr.1012	Retinol dehydrogenase 10	N	n.i.	n.i.	**Y**	n.i.	N	**Y**
38	Dr.5555	Cyclin A1	N	n.i.	n.i.	Y*	N	N	**Y**
**D. Occurrences > 0.15% in ZEBRAOV**									
5	Dr.47289	Metallothionein 2	n.i.	**Y**	N	N	N	N	N
10	Dr.31066	Heat shock protein 90-beta	N	**Y**	n.i.	**Y**	**Y**	Y*	Y*
11	Dr.729	Lactate dehydrogenase B4	N	N	n.i.	**Y**	**Y**	Y*	Y*
13	Dr.1043	ATP synthase, H+ transporting, mit.F0 complex, subunit c (subunit 9)	Y*	Y*	Y*	**Y**	**Y**	N	**Y**
16	Dr.989	Zgc:56419 similar to stress-associated endoplasmic reticulum protein 1	N	n.i.	n.i.	**Y**	**Y**	N	**Y**
21	Dr.13590	Transcribed locus, moderately similar to zygote arrest 1	**Y**	n.i.	N	**Y**	N	**Y**	N
24	Dr.29860	Transcribed locus	N	n.i.	n.i.	n.i.	n.i.	n.i.	n.i.
31	Dr.1428	Ribosomal protein S15a	N	N	n.i.	N	n.i.	**Y**	**Y**
32	Dr.7383	Zgc:73301 similar to cytochrome oxidase III	n.i.	n.i.	n.i.	n.i.	n.i.	n.i.	n.i.
34	Dr.29755	Wu:fe38a04 similar to RL31_HUMAN 60S ribosomal protein L31	N	n.i.	n.i.	n.i.	n.i.	N	n.i.
35	Dr.23788	Glutathione S-transferase pi	N	n.i.	n.i.	N	N	n.i.	**Y**
36	Dr.14015	Im:7140333 weakly similar to latrophilin; lectomedin-1	n.i.	n.i.	n.i.	**Y**	n.i.	N	**Y**
40	Dr.24903	Zgc:66168 similar to ubiquitin; 40S ribosomal protein S27a	**Y**	**Y**	Y*	**Y**	Y*	N	**Y**
42	Dr.26808	Ribosomal protein L10a	N	N	N	N	**Y**	**Y**	Y*
44	Dr.1335	Ribosomal protein L36	N	N	N	**Y**	**Y**	N	**Y**
46	Dr.6496	Ferritin, heavy polypeptide 1	**Y**	**Y** *	**Y**	**Y**	**Y** *	**Y**	**Y** *
47	Dr.1209	Signal sequence receptor, beta	N	n.i.	n.i.	**Y**	n.i.	N	**Y**
51	Dr.11308	Ribosomal protein, large, P0	N	n.i.	N	**Y**	n.i.	n.i.	Y*
52	Dr.4947	Cold inducible RNA binding protein	N	**Y**	n.i.	**Y**	**Y**	**Y**	**Y**
53	Dr.1334	Ribosomal protein S25	**Y**	n.i.	**Y**	**Y**	n.i.	**Y**	**Y**

*UniGeneBuild#87. The homologous UniGene clusters in other vertebrate species were found with UniGene tool or Blast search. Y, homologous UniGene cluster identified in the library. Y*, homologous UniGene cluster identified in the library with an occurrence >0.15%. N, the homologous UniGene cluster was not detected in the library. n.i., an homologous UniGene cluster was not recovered in UniGene. The presence of homologous clusters in at least four tetrapods or at least five compared vertebrate species are underlined. The vertebrates dbEST cDNA libraries accession numbers used for comparison were: ID.15587 for rainbow trout (*Oncorhynchus mykiss*); ID.15459 for Atlantic salmon (*Salmo salar*); ID.11967 for Fugu (*Takifugu rubripes*); ID.9909 for *Xenopus tropicalis*; ID.15096 for swine (*Sus scrofa*), ID.10029, ID.14142, and ID.1389 for mouse (*Mus musculus*); ID.4908, ID.10552, and ID.5611 for human (*Homo sapiens*).

**Table 5:** Comparison of zebrafish follicle protein repertoire deduced from SAGE with the protein repertoire isolated by proteomic analysis

**SAGE-tag**	Nbr*	**GenBank**	Cluster**	**Gene**	**Protein name**	**UniProt accession number**	Gel piece ***
***R1 or R1cr***							
CATGTAAGACATCC	226	BC045879	Dr.1109	*bactin2*	Beta-actin2	Q7ZVI7, Q6NWJ6	VII, 13
CATGTAAAACCAAA	108	AF057040	Dr.25213	*bactin1*	Beta-actin1		
CATGTTGATGAGGG	113	BC044190	Dr.729	*ldhb*	Lactate dehydrogenase B4	Q9PVK4, Q803U5	IX
CATGCATTTTCTAA	90	BM571854	Dr.19916	*zp2.3*	Egg envelope protein ZP2 variant B [zp2.3]	Q98SS2	VII-IX, 10, 12–15
CATGCATTTTCTAA	90	BC093133	Dr.23439	*zp2.4*	Zp2.4 protein	Q502R0	VII-IX, 10, 12–15
CATGAGAAACGTTT	77	BC067692	Dr.26977	*zp3b*	Zona pellucida glycoprotein 3b	O12989, Q6NW78	VI, 12
CATGGTGGTGTTGA	75	NM_131331	Dr.30322	*zp3*	Zona pellucida glycoprotein 3	Q9PWC7, Q6NW70	V, VI, 8–12
CATGTTAATAAAAG	53	NM_198809	Dr.5605	*tubb2*	Tubulin, beta, 2	Q6TGT0, Q6P5M9, Q6IQJ2	VI, 7
CATGTCTGTTTAAC	47	BC049058	Dr.11308	*rplp0*	Ribosomal protein, large, P0	Q7ZUG3, Q6P5K3	VIII, IX
CATGCTTAAATTGA	40	BC095386	Dr.30628	*gapd*	Gapdh protein (Fragment)	Q5XJ10	VIII, IX
CATGTTCTTTCTGT	36	BM778256	Dr.23435	*zp2.2*	Egg envelope protein ZP2 variant A [zp2.2]	Q98SS3	VII-IX, 10, 12–15
CATGCCACAGGGCA	35	CO917383	Dr.5705	*rps3*	Ribosomal protein S3	Q6TLG8	IX
CATGGCTGCCTTGG	34	NM_199795	Dr.23436	*tuba4l*	Tubulin alpha 3; Alpha tubulin-like protein	Q802Y6, Q6TGS5	V, 7
CATGAGGATCGAGG	30	BI844143	Dr.25678	*eno3*	Enolase 3, (beta, muscle)	Q568G3, Q6TH14	VI, 9, 11, 12
CATGTACAAGGCTT	28	AY394968	Dr.18315	*eef1g*	Elongation factor 1-gamma	Q6PE25, Q6NYN8	VI, 7, 8, 12
CATGTTCTCCCTGT	25	NM_001003844	Dr.28231	*rpl6*	60S ribosomal protein L6	Q6DRC6, Q567N5	X
CATGTGCTGAAAGT	16	NM_213058	Dr.26116	*hspa5*	Heat shock 70kDa protein 5	Q7SZD3, Q6P3L3	III
CATGCCCGGTGGAT	15	BC045841	Dr.24802	*hspa8*	Heat shock protein 8	Q7ZVJ1, Q6TEQ5, Q6NYR4	III
CATGGTGTAAGTGA	15	NM_212772	Dr.28850	*tuba8l*	Tubulin, alpha 2	Q6TNP9	V, 7
CATGCTCAAAGATG	12	BG305824	Dr.25868		Hypothetical protein zgc:64133 (L-lactate dehydro. activity)	Q7T334	IX
CATGTCTTCTGAAA	12	CO352070	Dr.34146	*zp3al*	Zona pellucida glycoprotein 3a, like	Q5TYP2	V, VI, 9–12, 14, 15
CATGTCTTCTGAAA	12	CO351980	Dr.25594	*zp3a*	Zona pellucida glycoprotein 3a	Q5TYP5	V, VI, 9–12, 14, 15
CATGTTTTTTTTGA	11	BC056719	Dr.24206	*cct4*	Chaperonin containing TCP1, subunit 4 (Delta)	Q6PH46, Q6P123	V, 8
CATGTCTTGGTTAA	9	NM_200153	Dr.6969		Hypothetical protein zgc:55944 (aminopeptidase I activity)	Q803B5	8
CATGTGTTGATGAA	8	NM_199547	Dr.3463	*ssb*	Sjogren syndrome antigen B (Autoantigen La)	Q7ZTI0	12
CATGTGAAGCACTT	8	NM_212718	Dr.18319		ZPA domain containing protein [si:dkeyp-50f7.2]	Q8JIX8, Q5TYX2	III
CATGAGAAGGAGGA	6	BC055516	Dr.20938	*sod1*	Superoxide dismutase [Cu-Zn]	O73872	22
CATGTCTGAGCTGA	4	AY398308	Dr.4751	*aldh2*	Mitochondrial aldehyde dehydrogenase 2 family [aldh2l]	Q6TH48, Q6NWJ9	10, 11
CATGAACCACTCAA	4	BC050953	Dr.7644	*cct2*	Chaperonin containing TCP1, subunit 2 (Beta)	Q6PBW6	V
CATGACACAAAATA	4	BQ263267	Dr.25009	*vg1*	Vitellogenin 1	Q8JH36, Q8JH37, Q90YN8	I-XI, 1–7, 10–17, 21–24
CATGTGCTCAAAAT	4	BC045933	Dr.134	*cct7*	Chaperonin-containing T-complex protein 1 eta subunit	Q8JHG7 Q7ZTS3	7
CATGATTGTATGGT	3	BM860549	Dr.7343		Acyl-Coenzyme A dehydrogenase, C-4 to C-12 straight chain	Q7ZTH8, Q6NYM4, Q502R3	VII, 12
CATGATGTGGATCC	3	CK673334	Dr.28241	*rpl7a*	Ribosomal protein L7a	Q6PBZ1	X
CATGCATTGTATAT	3	BM101604	Dr.11543		Zgc:103482	Q5XJA5	VI, 12, 13
CATGCCGACCCTAC	3	BC083399	Dr.11543		Zgc:103482	Q5XJA5	VI, 12, 13
CATGTTCTCCCTCA	3	BC091659	Dr.10133		Zgc:110766 (translation elongation factor activity)	Q5BJ17	12, 13
CATGAAGATGGAAA	3	BQ285089	Dr.24685		Hyp.prot.zgc:111961(hydro.-translo.F-type ATPase complex)	Q4VBK0	VI
CATGTTTTGTACAA	2	NM_200174	Dr.8999		Hypothetical protein zgc:55702	Q7ZVR7	8
CATGTCACAGGCCA	2	CO919203	Dr.29735	*naca*	Nascent polypeptide-associated complex alpha subunit	Q8JIU7	IX
CATGAAGCGGAGCT	2	BC059705	Dr.28202		Hypothetical protein zgc:73404 (Peptidase)	Q6NSN3, Q6PBH6	10
CATGCAAGTGTGAT	1	BI843130	Dr.20270		Wu:fi20c07 Hypothetical protein (Fragment) (Pentaxin)	Q7SZ53, Q5XJ77, Q4V8X3	17, 18
CATGCCTGATCTGT	1	NM_201052	Dr.1065	*rpsa*	Ribosomal protein SA	Q803F6	16, 20
CATGAGGGCTGGAA	1	NM_201290	Dr.6928	*cct6a*	Chaperonin containing TCP1, subunit 6A (Zeta 1)	Q7ZYX4	IV, 7, 8
CATGTTGAACTCCA	1	BQ419723	Dr.28864	*serpina1*	Novel protein similar to Alpha-1 antiproteinase (Serpina1)	Q5SPJ1, Q5SPJ4	10, 11
***R2 or R3***							
CATGGAGATCTCTA	2	BC044432	Dr.1051	*dldh*	Dihydrolipoamide dehydrogenase	Q803L1, Q6TNU6	8
CATGTGTGCACGCA	1	BC095354	Dr.39983		Hypothetical protein zgc:110655	Q503F1	IX
***Rn1 or Rn2***							
CATGTTAAAACGTG	11	CA471093	Dr.7108	*hspd1*	Hspd1 protein (Heat shock 60 kD protein 1)	Q803B0	IV
CATGCTGAACGGAT	5	BC073176	Dr.5529		GDP dissociation inhibitor 3;GDP dissociation inhibitor 2	Q7ZVL9, Q6TNT9	VI, 11
CATGCACATCAGAT	1	CK400766	Dr.11310	*tuba1*	Tuba1 protein	Q8AWD6, Q6NWK7, O42271	V, 7–9, 23
CATGATGAGACAAA	1	CO917509	Dr.6501		Sb:cb825 protein (Fragment)	Q803D7	V, 10
CATGCAAGTGTGTC	1	BC053271	Dr.28298	*cct3*	Chaperonin-containing TCP-1 complex gamma chain	Q8JHI7, Q7T2P2	IV
***R1 or R1cr, multiple matches***							
CATGAAAAAAAAAA	153	BI879427	Dr.296	*ckb*	Creatine kinase, brain	Q7ZU04, Q8AY63, Q5RIR2	VII, 12–15
CATGACATCACATT	60	AY394971	Dr.664	*tuba8l4*	Similar to tubulin, alpha 1 (tubulin, alpha 8 like 4)	Q7ZVI6, Q6NWJ5	V, 7–9, 23
CATGGCCATTGAGG	4	BI671108	Dr.20576		Mitochondrial ATP synthase alpha subunit	Q6EE37	V, 7
CATGAAGAAAAAAA	3	AF254638	Dr.31985	*vg3*	Vitellogenin 3 (Fragment) [vg3]	Q9DFT9	II, VIII, 6, 21
CATGATCACTGGAA	3	BM182705	Dr.15108		LOC407641 protein (Fragment) translation elongation factor	Q7ZWA1	VI
CATGTATAAATTCC	2	NM_001002374	Dr.31390		Zgc:92082	Q6DHT4	8, 9
CATGTGTGTGTGAG	1	BG304242	Dr.7952	*pkm2*	Pyruvate kinase, muscle	Q7ZVT2, Q6NXD1	7, 8, 9
CATGCAGGAGTTCA	1	CA496254	Dr.4724	*eno1*	Enolase 1, (Alpha) [zgc:73152]	Q6IQP5	8, 9, 11, 12
***Non detected***							
CATGTGGCTTTCAA	0	NM_200446	Dr.831	*chia*	Eosinophil chemotactic cytokine	Q7SYA8, Q6TH31	IV
CATGCTGGACCAGC	0	NM_131804	Dr.10788	*nots*	Nots protein	Q5PR42, Q9DDE2	16, 20

*Nbr, is the number of times a tag was counted in ZEBRAOV. **Zebrafish UniGene Build#87. ***SDS-PAGE slices numbered I to XI or two-dimensional PAGE excised spot area numbered 1 to 24 (see Figure 4).

**Table 6:** Comparison of zebrafish follicle transcript occurrence of proteins identified by proteomic analysis, with the corresponding protein repertoire deduced from transcripts expressed at over 0.15% of the mRNA population in human ovary cDNA libraries

Zebrafish*	Human**	**Human gene**	**Protein name**	**Zebrafish**			**Human**		
				**SAGE**	**ID.9767**	**ID.15519**	**ID.4908**	**ID.5611**	**ID.10552**
Dr.1109	Hs.520640	*ACTB*	Beta-actin	**0.822**	**0.317**	**0.752**	**0.404**	**0.503**	**0.535**
Dr.25213	Hs.520640	*ACTB*	Beta-actin	**0.393**	**0.326**	**0.698**	**0.404**	**0.503**	**0.535**
Dr.729	Hs.446149	*LDHB*	Lactate dehydrogenase B	**0.411**	0.035	0.046	**0.210**	0.140	**0.197**
Dr.5605	Hs.433615	*TUBB2*	Tubulin, beta, 2	**0.193**	**0.290**	**0.852**	**0.194**	0.140	**0.159**
Dr.11308	Hs.448226	*RPLP0*	Ribosomal protein, large, P0	**0.171**	0.079	0.023	**0.381**	**0.199**	**0.384**
Dr.30628	Hs.544577	*GAPDH*	Glyceraldehyde-3-phos. dehydro.	0.145	0.096	0.116	**1.105**	**1.018**	**0.816**
Dr.5705	Hs.546286	*RPS3*	Ribosomal protein S3	0.127	0.017	0.008	**0.708**	**0.889**	**0.356**
0Dr.18315	Hs.444467	*EEF1G*	Elongation factor 1-gamma	0.101	0.052	0.089	**0.700**	**0.304**	**0.525**
Dr.28241	Hs.558380	*RPL7A*	Ribosomal protein L7a	0.011	0.088	0.004	**0.591**	0.081	**0.366**
Dr.1065	Hs.558354	*RPSA*	Ribosomal protein SA	0.003	0.088	0.008	**0.342**	**0.187**	0.103
Dr.7952	Hs.198281	*PKM2*	Pyruvate kinase, muscle	0.003	0	0.017	**1.035**	**0.374**	**0.657**
Dr.4724	Hs.517145	*ENO1*	Enolase 1	0.003	0	0	**2.023**	**0.573**	**0.582**

*Zebrafish UniGene Build#87. **Human UniGene Build#187. The values indicated are the percentages of species specific UniGene clusters as evaluated by the percentages of corresponding tags or EST counts in the mRNA population identified in ZEBRAOV SAGE tag database and dbEST libraries. Percentages at over 0.15% were bolded.
